# An integrated resource for ischemic heart disease defines hallmarks and heterogeneity across time and space

**DOI:** 10.1016/j.apsb.2025.11.020

**Published:** 2025-11-14

**Authors:** Tianhao Wang, Yining Hu, Wenbo Guo, Haoran Li, Menglei Wang, Bojin Chen, Hudong Bao, Meng Gao, Xiang Li, Qian Chen, Minjie Shen, Xin Shao, Jie Liao, Xiaohui Fan

**Affiliations:** a State Key Laboratory of Chinese Medicine Modernization, College of Pharmaceutical Sciences, Zhejiang University, Hangzhou 310058, China; b Zhejiang Key Laboratory of Chinese Medicine Modernization, Innovation Center of Yangtze River Delta, Zhejiang University, Jiaxing 314100, China; c Jinhua Institute of Zhejiang University, Jinhua 321016, China; d Hangzhou Medical College, Hangzhou 311399, China; e Hangzhou TCM Hospital Affiliated to Zhejiang Chinese Medical University, Hangzhou 310007, China

**Keywords:** Ischemic heart disease, Single-cell atlas, Spatial transcriptomics, Gene program, Knowledge graph, Interactome

**To the editor:**

Ischemic heart disease (IHD), a leading cause of mortality worldwide, is primarily caused by atherosclerosis. Currently, the pathology of IHD is still not fully understood. Decades of pharmacology research have accumulated a wealth of knowledge on genetic pathology, but conventional approaches cannot resolve tissue microstructures and cell dysfunctions^1^. Single-cell RNA sequencing (scRNA-seq) and spatial transcriptomics (ST) paved new roads for IHD research. However, technical limitations and inadequate sample volume still hindered understanding of cells and tissue architecture at different stages of IHD^2^. Furthermore, the inconsistency of experimental operations and computations between laboratories make cross-validation of different studies less reliable. In this letter, we introduced Spatial Single-cell Ischemic Heart Disease Browser (ssIHDB), a comprehensive, spatio-temporally resolved resource that integrated single-cell and spatial transcriptomes with a manually curated knowledgebase of genes, drugs, and comorbidities relevant to IHD (https://xomics.com.cn/ihdb/) (Supporting Information Fig. S1).

## 1 IHDAtlas resolves cellular heterogeneity of pathological fibrosis across temporal niches

**Figure 1:**
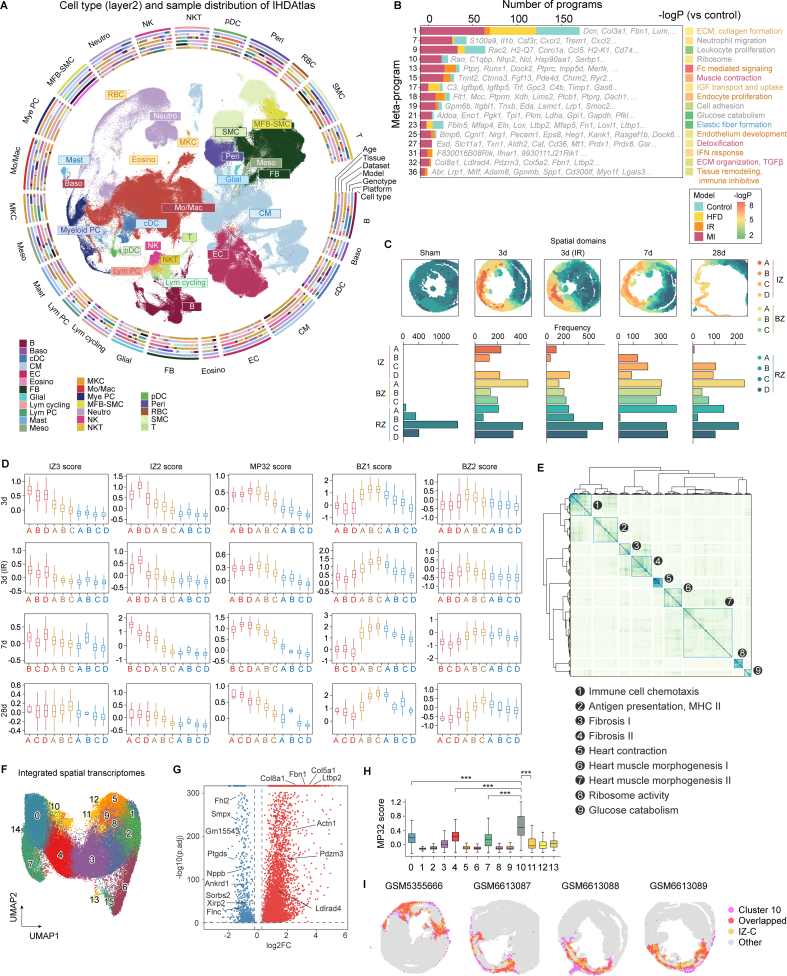
Integrative single-cell and spatial analysis substantiated a specific fibrosis region crowded by *Ltbp2*-hi cardiomyocytes. (A) A mouse single-cell IHD atlas composed of 40 major cell types, with meta information, including cell type composition, technical platform, genotype, model, dataset, and age quantified by the concentric bands; (B) Meta-program (MP) analysis uncovered 16 MPs that were significantly activated or suppressed after MI, IR injury, or atherosclerosis; (C) Spatial domains of ST samples (upper) and number of spots belonging to each domain (bottom); (D) Scores of BZ1, BZ2, MP32, IZ3, and IZ2 in each domain; (E) Spatial MP analysis uncovered 9 MPs from mouse MI samples; (F) Integrated spatial transcriptome atlas; (G) Volcano plot of DE genes in cluster 10; (H) Predicted MP32 scores of spot clusters (∗∗∗*P* < 10^−16^); (I) Comparison of spatial distribution pattern of cluster 10 and IZ-C in representative samples.

To enable comparison and cross-validation of cells from different studies, we integrated scRNA-seq data of wild-type mouse models from public resources and tested multiple integration tools and found that scVI most competently removed batch effects and preserved developmental relationships (Supporting Information Fig. S2A, and Tables S1 and S2)^3^. By clustering at a broad range of resolutions, we identified 6 major cell categories (layer 1), 24 minor categories (layer 2), and 40 basic cell types (layer 3) (Fig. 1 and Fig. S2B). Gene expression and label transfer demonstrated that the annotation was accurate with minimal batch bias (Supporting Information Figs. S2C and S3A). Notably, the atlas unbiasedly defined rare cell types, such as MKCs, mast cells, and lymphocyte progenitors, that usually cannot be defined in a single dataset (Fig. S3).

Next, the basic cell types were further divided into functional subtypes (layer 4) to test whether the integration kept fine cellular heterogeneity. Fibroblast (FB) is one of the most abundant populations and plays essential roles in cardiac repair^4^. FBs were first clustered into 15 subtypes, which recovered 13 FB subtypes previously reported in multiple myocardial infarction (MI) studies, including *Comp*^hi^ matrifibrocyte abounding at 14 day-post-MI (DPI), *Acta2*^hi^*Cthrc1*^hi^ myofibroblast (MFB) abounding at 7 DPI, *Cilp*^hi^*Meox1*^hi^ activated FB mainly from 3 day-post-ischemia reperfusion (IR), a *Cxcl5*^hi^*Ccl2*^hi^ cluster abounding at 1 day-post-IR, a Wnt-related cluster, a cycling cluster mainly from 3 DPI, a *Timp*^hi^ cycling intermediate (CI) cluster, an *Fgl2*^hi^*Ccl19*^hi^ transitional cluster, an *Ly6c1*^hi^*Ly6a*^hi^ F-SH cluster, a *Gsta3*^hi^*Cxcl14*^hi^ F-SL cluster, an unfolded protein responding *Gdf15*^hi^ cluster, a specific extracellular matrix (ECM) remodeling *Efhd*^hi^ cluster from 42 DPI, and a profibrotic *Col4a4*^hi^ cluster abounding at 90 DPI (Supporting Information Table S3 and Fig. S2C)^5^^,^^6^. Fine clustering discovered that the CI cluster could be further divided into a *Ccnb2*^lo^*Aunip*^hi^ cluster, a deactivated cluster, and two *Ccnb2*^hi^ clusters (layer 5). Besides, the MFBs could also be subclustered into a pro-inflammatory *Tnxb*^hi^ cluster, a *Matn4*^hi^ cluster related to vascular morphogenesis, and a typical MFB cluster. This atlas also defined several previously unknown FB clusters, including a metal ion-responding *Sepp*^hi^ cluster, and an HFD-responding cluster expressing profibrotic genes *Rbp4*, *Gdf10*, and *Smoc1* (Supporting Information Table S4). Three clusters were removed because these were mainly from a single sample or dataset. Besides, the atlas well distinguished other functional subpopulations such as endothelial cell (EC) subtypes (arterial, capillary arterial, capillary venous, large venous, lymphatic, and endocardial ECs), neutrophils at different stages (myeloblasts, metamyelocytes, myelocytes, banded neutrophils, and mature neutrophils), and progenitors. These results demonstrated that the integration successfully resolved cellular heterogeneity and could generate biologically meaningful conclusions.

Smooth muscle cells (SMCs) play essential roles in ECM remodeling. Except for the artery or aortic SMCs, a specific population that mimics the previously poorly defined myofibroblast-like SMC (MFB-SMC) was discovered. As reported this population was deprived of classical SMC markers (*Myh11*, *Smtn*) but expresses *S100a4*, vascular SMC markers (*Sncg*, *Sdc1*), myofibroblast markers (*Dkk3*, *Cthrc1*), and unique markers including *Anxa8*, *Rarres1*, *Tnfrsf11b*, and *Mmp3* (Fig. S3D)^7^. Most of these cells came from mice after 12–26 weeks of high fat diet (HFD) (Fig. S3E and S3F), and might prompt fibril formation and calcification, which suggested that these cells participated in aorta atherosclerosis (Fig. S3G).

## 2 Integrative analysis of single-cell and spatial atlases revealed a fibrotic region crowded by *Ltbp2*^hi^ CM

NMF is powerful to delineate cellular activities from high-throughput sequencing data and generate insightful gene sets. However, the accuracy of NMF analysis is sensitive to data volume, batch effects, and computation methods. To address these challenges, we adopted a modified meta-program (MP) analysis to leverage the large sample volume^8^. We first applied this strategy to mouse single-cell samples as the amount of available single-cell samples greatly exceeded ST (Supporting Information Methods and Materials). This generated robust programs and 16 program clusters significantly associated with IHD (Fig. 1B and Supporting Information Tables S5-S7). Collectively, we found 4 MPs related to tissue remodeling (MP1, MP25, MP32 and MP36), among which collagen formation (MP1) was enriched in HFD model, and MP32 was significantly enriched in both MI and IR. Covariate analysis revealed that cluster 32 was mainly composed of programs from 7 DPI (Fig. S3H and Table S8). Interestingly, cardiomyocytes recurrently contributed to the cell population with the highest MP32 levels in scRNA-seq samples (Supporting Information Fig. S4). In the atlas, the MP32 score was highest in a fibrotic subtype of CM highly expressing *Ltbp2*, a gene encoding a latent transforming growth factor binding protein. The majority of *Ltbp2*^hi^ CMs appeared to originate from 3 days after IR injury or MI (Fig. S3I and S3J). Of note, FBs and the MFB-SMCs had relatively low MP32 scores, which distinguished MP32 from classical fibrosis activity.

To better portray the characteristics and functions of MPs, we scored MPs associated with ischemic injury in a ST sample series from sham to 28 DPI models (Fig. S3A). All MPs exhibited spatio-temporal heterogeneity. MP15 related to muscle contraction decreased significantly within IZ at 4 h post-MI, and MP27 (detoxification) was activated at the IZ/BZ border at 1 DPI and within IZ at 3 DPI. At 3 DPI, MP32 was significantly activated, and MPs related to tissue remodeling and immune inhibition were activated in IZ. Surprisingly, only MP32 retained high activeness in whole IZ at 28 DPI (Supporting Information Fig. S5A).

To define the spatial heterogeneity of the infarcted myocardium, we used Bayesian Analytics for Spatial Segmentation (BASS) to identify spatial domains on data from sham, 3, 7, and 28 DPI. This produced 12 domains from infarcted zone (IZ), border zone (BZ) to remote zone (RZ) expressing reported markers of IZ, BZ, and RZ (Fig. 1C)^9^. One domain was removed due to its low spot number. Differential expression (DE) analysis revealed that IZ-B corresponded to the reported IZ2, and the combination of IZ-A and IZ-D corresponded to the reported IZ3. IZ-C did not correspond to reported zones. Consistent with previous studies, the total area of IZ-A and IZ-D decreased from 18.8% of all spots at 3 DPI to 3.8% at 7 DPI^9^. Of note, the MP32 score was highest in BZ-A at 3 DPI but gradually spread to IZ-C over time, indicating that MP32 was first activated in BZ and spread to IZ later (Fig. 1D). DE analysis revealed that IZ-C highly expresses ECM fibrosis, and Tgf*β* related genes (Fig. S4C). Deconvolution of CM subtypes from a 7 DPI sample to ST data showed the MP32^hi^ CM was correlated to the MP32 score and mainly came from IZ-C (Fig. S5B and S5C), while other cell subtypes were clearly located to other zones (Figs. S6C and S7).

To further verify that IZ-C closely related to cardiac fibrosis, MP analysis was performed on all mouse ST samples. This obtained 9 spatial MPs (SMPs) (Fig. 1E). SMPs successfully recapitulated functional activity across different MI samples, including immune cell chemotaxis (SMP1), and heart morphogenesis (SMP6, SMP7) (Supporting Information Table S9). Many SMPs were also observed in single-cell MPs, such as collagen formation (MP1, Jaccard similarity = 0.523) and glucose catabolism (MP21, Jaccard similarity = 0.338) (Supporting Information Table S10). SMP3 and SMP4, involving TGF binding and fibrosis genes, resembled single-cell MP32 (Fig. S5D). Then, we adopted a different strategy that integrated all mouse ST samples and divided spots into 16 clusters based merely on transcriptomes (Fig. 1F). Clusters 14 and 15 were excluded because of low numbers of spots. MP32 activeness was significantly higher in cluster 10, while other IZ and BZ markers were less expressed (Fig. 1G and H). Spatial distributions of cluster 10 in ST samples also mirrored IZ-C in the same samples (Fig. 1I), which confirmed that the cluster 10 corresponds to IZ-C. This demonstrated that a fibrotic zone could be recurrently discovered by integrative spatial analysis. As IZ-C was independent of reported zones, we named it “fibrosis zone” (FZ) for its close relation to the fibrogenic MP32. We reasoned that FZ was missing from previous studies because CMs were usually not considered as functional participants within infarcted myocardium.

## 3 SsIHDB web portal facilitates reuse of single-cell and spatial omics data and seamless integration with pharmaceutical analysis

Reusing existing scRNA-seq datasets can assist in validating IHD-related scientific findings. SsIHDB provides an out-of-the-box and feature-rich web portal for fast reuse of single-cell and spatial samples (Supporting Information Fig. S8). In brief, ssIHDB visualize samples with high degrees of freedom, such as various cell layouts for heatmap, CCI scores, or spatial deconvolution. We provided a manual of ssIHDB in Supporting Information (Supporting Information Supporting manual of ssIHDB). The data used by ssIHDB was derived from a coherent analysis pipeline based on our atlases (Supporting Information Fig. S9) with cell type annotation and deconvolution models trained on the IHDAtlas cell annotation. Furthermore, ssIHDB provides an online DE analysis module, where users can select spots of interest and perform *t*-test on all genes.

To better utilize experimentally verified IHD-related genes in high-throughput transcriptome analysis, drug repurposing, and comorbid analysis, we manually curated 6035 genes, 3157 small-molecule or biotech drugs, and 79 comorbidities that have been reported to be associated with IHD (Supporting Information Table S11). All genes were annotated by ontology and experimental details retrieved from the original studies (Supporting Information Fig. S10A–S10D). Molecular interactions including 136,020 protein–protein interactions, 5625 miRNA–target interactions, and 5635 drug-target interactions, and 2275 gene-comorbid associations were collected from functional annotation databases and published articles (Methods). Basic statistics of ssIHDB is provided in Supporting Information Fig. S11. Just for humans, these data constitute a large IHD knowledge graph (IHDKG) with 12,810 nodes and 89,596 edges (Fig. S10G). Furthermore, we integrated DE information from single-cell analysis into IHDKG. Based on IHDKG, ssIHDB provides a network analysis tool to integrate single-cell and spatial transcriptomics with pharmacological analysis (Fig. S10C–S10F). For example, ssIHDB provides one-click DE network construction for every single-cell and spatial sample. Besides, users can input gene lists into the search box and ssIHDB will automatically match all IHD-related genes and construct a network with their neighbors (Supporting Information Supporting manual of ssIHDB).

In summary, this study reported ssIHDB, a comprehensive knowledgebase designed for IHD that resolves single-cell and spatio-temporal heterogeneity. The hierarchical cell type annotation of IHDAtlas defined disease-specific cell subpopulations at unprecedented resolution. Furthermore, integrative analysis of atlases first manifested a shifting fibrosis area crowded by *Ltbp2*^hi^ CMs with TGF binding activity. These results imply that CM was a potential target for anti-fibrotic therapy. We developed a ssIHDB web portal to deposit, reuse, visualize, and perform online analysis on public single-cell and spatial data and made all results of this study available on the website with a coherent annotation.

## Author contributions

Xiaohui Fan and Jie Liao conceived the study. Tianhao Wang and Yining Hu collected the datasets. Tianhao Wang, Yining Hu, and Wenbo Guo preprocessed scRNA-seq and spatial transcriptomics datasets. Haoran Li, Menglei Wang, Bojin Chen, and Hudong Bao collected and curated the gene, drug, pathway, and miRNA information, respectively. Meng Gao performed downstream analysis of scRNA-seq data. Xiang Li and Qian Chen provide professional biological guidance. Minjie Shen imported the tables into the database. Xin Shao performed network analysis. Yining Hu and Tianhao Wang drafted the manuscript. Tianhao Wang performed the integration, designed the website and analyzed all data. All authors approved the final version of the manuscript.

## Data availability

SsIHDB is an open-source database that any feature on the web is freely accessible. All scripts used for construction and downstream analysis of IHDAtlas in this manuscript can be found on GitHub (https://github.com/SpaTrek/IHDB/).

## Conflicts of interest

The authors declare no competing interests.

## Appendix A Supporting information

The following are the Supporting Information to this article:
