# Supplementary material for: An integrated resource for ischemic heart disease defines hallmarks and heterogeneity across time and space
Source: Acta Pharm Sin B. 2025 Nov 14;16(1):642–6. doi: 10.1016/j.apsb.2025.11.020 (PMC12828080; doi:10.1016/j.apsb.2025.11.020)
Supplement: Multimedia component 1 [file mmc1.pdf]

Supporting information for

Letter to the editor

An integrated resource for ischemic heart disease defines hallmarks and heterogeneity across time and space

Tianhao Wang[a,b,e,†], Yining Hu[a,b,c,†], Wenbo Guo[a,b,e,†], Haoran Li[a,b,e], Menglei Wang[a,b,e], Bojin Chen[a,b,e], Hudong Bao[a,b,e], Meng Gao[a,b,e], Xiang Li[a,d], Qian Chen[a,b,e,f], Minjie Shen[a,b,e], Xin Shao[a,b,e,f,*], Jie Liao[a,b,e,*], Xiaohui Fan[a,b,c,d,e,f,*]

[a]*College of Pharmaceutical Sciences, Zhejiang University, Hangzhou 310058, China*

[b]*State Key Laboratory of Chinese Medicine Modernization, Innovation Center of Yangtze River Delta, Zhejiang University, Jiaxing 314100, China*

[c]*Jinhua Institute of Zhejiang University, Jinhua 321016, China*

[d]*Hangzhou Medical College, Hangzhou 311399, China*

[e]*Zhejiang Key Laboratory of Chinese Medicine Modernization, Innovation Center of Yangtze River Delta, Zhejiang University, Jiaxing 314100, China*

[f]*The Joint-Laboratory of Clinical Multi-Omics Research between Zhejiang University and Ningbo Municipal Hospital of TCM, Ningbo Municipal Hospital of TCM, Ningbo 315100, China*

Received 8 September 2025; Received in revised form 23 October 2025; accepted 7 November 2025

*Corresponding authors.

E-mail addresses: fanxh@zju.edu.cn (Xiaohui Fan), liaojie@zju.edu.cn (Jie Liao), xin_shao@zju.edu.cn (Xin Shao).

† These authors made equal contributions to this work.

1. Materials and methods

1.1. Collection of omics data

Single-cell and ST studies were searched from PubMed using keyword combinations of ‘single-cell’, ‘ischemic heart disease’, ‘myocardial infarction’, ‘scRNA-seq’, ‘spatial’, ‘spatial transcriptomics’, ‘coronary’, ‘angina pectoris’, and ‘ischemic cardiomyopathy’. The corresponding scRNA-seq and ST datasets were downloaded from GEO, ArrayExpress, figshare, Zenodo, and Single Cell Portal databases. Description of the datasets is accessible on the ssIHDB website and Supporting Information Table S1.

1.2. Collection of conventional data

IHD-related literature was obtained from PubMed by using Entrez Direct[1]. The ‘esearch’ function was used to search for articles with terms ‘coronary disease AND English [LANG] AND has abstract [FILT] NOT review [PTYP]’. The ‘efilter’ function was used to filter for articles published later than the year 2000. The ‘efetch’ and ‘xtract’ function were used to obtain PMID and abstract of the articles by ‘efetch -format xml | xtract -pattern PubMedArticle -element PMID AbstractText’. In sum, 157,249 articles were found for further text mining. Python package nltk (version 3.7) was used for tokenization and data cleaning[2]. After that, standard gene symbols were obtained from NCBI database (updated on April 30, 2023, https://www.ncbi.nlm.nih.gov/) to match protein-coding gene symbols within abstract. Non-protein coding transcripts such as miRNA, lncRNA, and circRNA were matched with LNCipedia (version: 5.2, https://lncipedia.org/)[3], miRBase (https://www.mirbase.org/)[4], and circBank (http://www.circbank.cn/)[5], respectively. Finally, 7,249 non-repeated gene symbols or transcripts were found, and were manually verified by reading the corresponding articles. Information on how these genes participate in IHD pathogenesis, including relations by influencing drug action, sexual distinction, and sponge regulation, were recorded. Meanwhile, information on research design and supporting evidence were recorded. Abbreviations and full names of comorbidities are listed in Supporting Information Table S11.

IHD-related drug information was obtained from DrugBank database (version: 5.1.10, https://go.drugbank.com/) for text mining[6]. After manual confirmation, a total of 3,157 drugs were obtained, of which description or citations were matched with ‘coronary artery disease’ or the comorbidity terms. Drug name, DrugBank ID, CAS

number, UNII, synonyms and target gene information were recorded.

1.3. Collection of molecular relations and gene-disease associations

Human and mouse PPIs were obtained from STRING database (version: 11.0, https://string-db.org/)[7]. Interaction pairs with combined score > 0.4 were retained. MTIs were obtained from miRTarBase (version: 9.0 beta, https://mirtarbase.cuhk.edu.cn/)[8]. CeRNA networks for lncRNA and circRNA were obtained from lncACTdb (version: 5.2, http:// LncACTdb 3.0 : Home (hrbmu.edu.cn) /) and manual curation[9]. GDAs were obtained by manual curation. Classical Pathways information was obtained from Kyoto Encyclopedia of Genes and Genomes (KEGG) database (version 106.0, https://www.kegg.jp/).

1.4. Preprocessing of scRNA-seq data and ST data

All scRNA-seq samples were transformed into AnnData objects before analysis using Scanpy (v1.9.3). Samples with less than 150 cells were discarded. Demultiplexing of hashtagged samples (Table S1) was performed by 'hashsolo' function from Scanpy[10]. In this study, median absolute deviance (MAD) was used to measure the dispersion of a metric over a scRNA-seq dataset. MAD was defined by $MAD = median(|X_i - median(X)|)$ with X_i being a metric of a cell and $median(X)$ compute the median value of the metric over all cells. Low quality cells were defined by the following criteria: (1) any of the logarithmic number of total counts, logarithmic number of expressed genes, and the ratio of counts in top 20 genes > 5 MADs. (2) considering various cell types were involved, cells with the ratio of mitochondrial counts > 5 MADs but not < 5% were defined as damaged cells[11]. Sequencing doublet was excluded using the scDblFinder package (v1.18). Ensembl IDs were transformed into gene symbols by using GRCh38.p14 (release 113) and GRCm38.p6 (release 102) primary assemblies. External RNA Controls Consortium (ERCC) reads, Hashtags, and exogenic genes including DsRed, ZsGreen1, EGFP, YFP and tdTomato were removed. Data normalization and centering was performed for each sample[12].

1.5. Integration and annotation of scRNA-seq mouse IHD atlas

The core single-cell atlas of mouse IHD landscape is composed of samples without drug or gene perturbation or with perturbations merely for IHD modeling. A total of 421 samples were integrated by using scVI, harmony, BBKNN, and Scanorama,

separately, with sample labels as batch keys. ScVI was set (n_layer=2, n_latent=60, gene_likelihood='nb'), neighbor distance matrix and neighborhood graph were calculated using 'pp.neighbors' function (n_neighbors=15, metric='cosine') using all scVI low-dimensional representations. Harmony integration was performed by running the 'IntegrateLayers' function (method=HarmonyIntegration, orig.reduction='pca') on normalized, scaled expression matrix of top 5000 highly variable genes[13]. Cell type clustering and manual annotation was done by Leiden clustering at a wide range of resolution on scVI neighbor graph.

1.6. Sample-wise analysis of scRNA-seq data

After cells and genes filtration, samples were either analyzed individually or integrated into large datasets. For coherent annotation of cell types, we first trained three models using the layer 1–3 of the mouse atlas annotation system by CellTypist (v1.5.3)[14]. After that, we used layer3 for automatic annotation for mouse data. Human cells were annotated with the Celltypist official models 'Healthy Adult Heart' and 'Immune All High'. Then, cell subtypes were defined at the resolution of 0.3 for all cell types with over 150 cells using the 'FindClusters' function from Seurat (v5.0.1). DEGs of subtypes were found using DESeq2 (v1.38.3) applied with zinb-wave recipe (v1.28.0) as recommended by Nguyen et al.[15]. Briefly, 'SummarizedExperiment' object was created from counts matrix, 'zinbwave' function was used to compute observational weights (K = 2, epsilon=1e12, observationalWeights = TRUE), and the count matrix of the output was added with pseudo-counts of one to get better performance[15]. Then, the 'DESeqDataSet' object was created and DESeq2 regression was performed by running 'DESeq' (sfType='poscounts', useT=TRUE, minmu=1e-6), followed by 'lfcShrink' to get comparison results. NMF was performed on the centered log-normalized matrix by the 'nmf' function (rank=30, method='snmf/r', seed='nndsvd') from NMF package (v0.27). For CCI inference, we performed the liana (v0.1.9) implementation of CellphoneDB with LR information from CellTalkDB[16-18]. For the convenience of online visualization, only entries with 'cellphone_pvals' under 0.05 and 'lr_means' ranked the top 1000 were kept. TSNE, UMAP, DPT and igraph layouts ('ForceAtlas2', 'Fruchterman Reingold', 'Kamadi Kawai', and 'Distributed Recursive Layout') were implemented using Scanpy. Single-cell GSEA was performed by GSDensity (v1.5.0) with Gene Ontology (GO) and Reactome gene sets. In brief, the gene sets were downloaded from MSigDB (v2023.2)[19], the differential distribution of gene set from

whole transcriptome was measured by KL-divergence by 'compute.kld' with default params. The difference threshold was set to 0.001 and probability scores were calculated for gene sets under the threshold.

1.7. Dataset-wise analysis of scRNA-seq data

Sample integration was performed by scvi-tools (v1.0.2)[20]. Details of processing pipeline are available in Supporting Information Fig. S9.

1.8. Generation of MPs

MPs were generated using scripts provided by Gavish et al.[12] with slight modifications. In brief, robust programs were programs that occurred repeatedly in different samples but not the same samples. Then, robust programs were clustered based on their shared top-ranked genes. An MP was defined as a set of genes that were shared by most programs from a cluster. Robust programs from pharmaceutically or genetically perturbed samples, and single-cell MPs primarily from a single dataset or contain less than 5 robust programs were removed. After that, covariate analysis was performed on the model proportion of each cluster to find single-cell MPs significantly associated with disease models. We compared the proportion of models (healthy, MI, HFD, and IR) of each single-cell MP to the reference proportion from the whole core atlas, then compared each model to the healthy model with the same reference by Chi-square test or Fisher's exact test. Those MPs with $P<0.05$ were enriched by GO, and Reactome pathways. Enrichment analysis was performed by ClusterProfiler (version 4.10.1)[21]. For spatial MPs, due to few samples available, a modified script was used to obtain MP clusters and genes. In brief, spatial robust programs were obtained using the same method as single-cell MPs, followed by hierarchical clustering of robust programs by Jaccard's similarity using 'pheatmap' (clustering_method = 'ward.D', cutree_rows = 10, cutree_cols = 10) from pheatmap package (v1.0.12). Comparison of single-cell and spatial MPs was done by hierarchical clustering of Jaccard's similarities between single-cell MPs and spatial MPs.

1.9. Spatial transcriptomics analysis

ST samples with the least complete data structure, *i.e.*, containing at least a high-resolution image, a raw count matrix, and a spot location table, were kept for preprocessing using SCTransform from Seurat. Preprocessing of ST samples was

similar to single-cell samples. The scoring of ischemic zone, border zone, and MPs were performed by the 'AddModuleScore' function from Seurat. Spatial domain analysis was performed by BASS according to Hu et al.[22] Cell composition deconvolution was performed by cell2location model train on the IHDAtlas reference single-cell atlas using default parameters[23]. Integration of all ST samples was performed according to Kuppe et al.[24]. In brief, all preprocessed samples integrated by harmony using the top 4000 HVGs across all slices. Then, adjacency matrix was computed using 'FindNeighbors' function from Seurat (dims = 1:30) and 'RunUMAP' was performed using all 50 harmony metrics. Spots were clustered using 'FindClusters' function (resolution = 0.6). Two clusters (14 and 15) were removed due to too few spots. DEGs of each cluster was identified by using 'FindAllMarkers' function (assay = 'SCT') following 'PrepSCTFindMarkers' from Seurat. Spatial NMF and robust program analysis were performed by the same scripts as single-cell NMP. Spatial MP analysis was performed using modified script of single-cell MP analysis.

1.10. Network visualization of IHDKG

The knowledge graph was visualized by Gephi (v0.10.1)[25].

2. Supporting tables

Tables S1–S11 are provided as separated Excel files.

3. Supporting figures

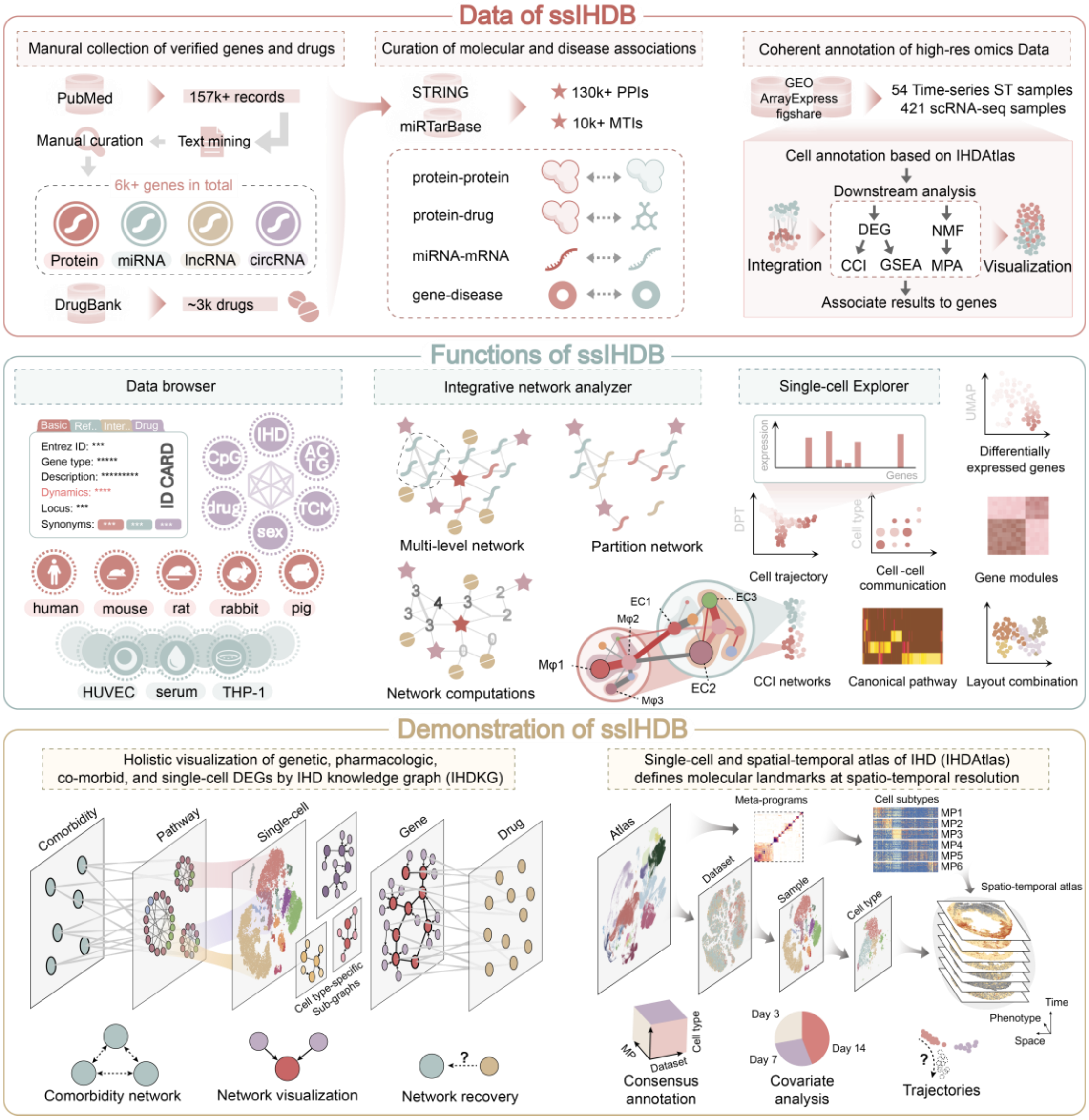

Figure S1 Overview of data coverage, presentation, and demonstration of ssIHDB. The top box includes the data collection and analysis procedure. The middle box shows the 3 functional modules of ssIHDB website. The bottom shows the coverage of the knowledgebase. DEG, differentially expressed gene. NMF, non-negative matrix factorization. CCI, cell–cell interaction. HUVEC, human umbilical vein endothelial cell.

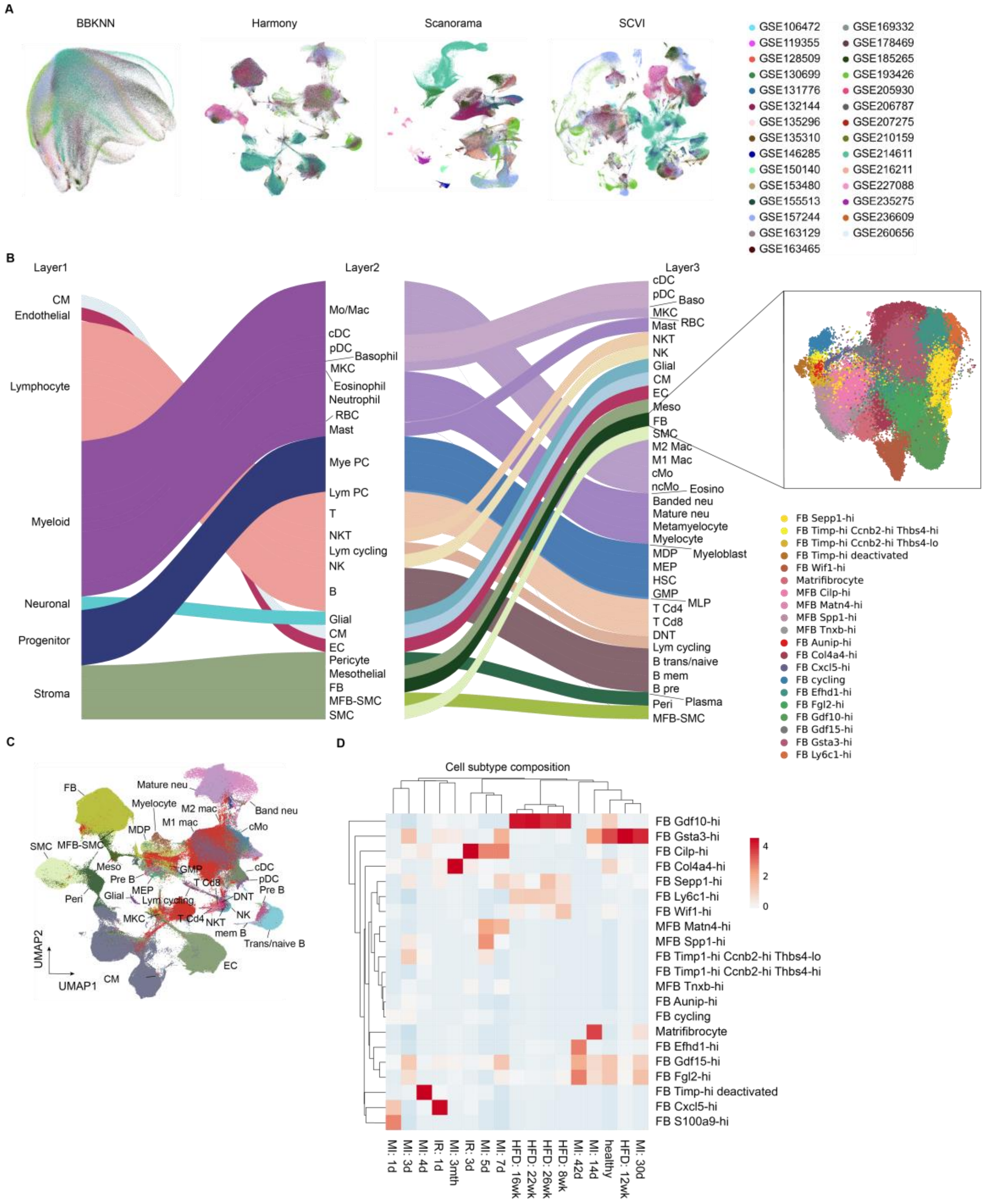

Figure S2 Integration of single-cell reference atlas and hierarchical cell type annotation. (A) Benchmark of integration algorithms for IHDAtlas; (B) The first three layers of annotation, which divides all cells into 40 common cell types, and clustering of 20 fibroblast subtypes at fine resolution; (C) Transferred cell type annotation to the Harmony integration; (D) Model composition of each fibroblast subtype.

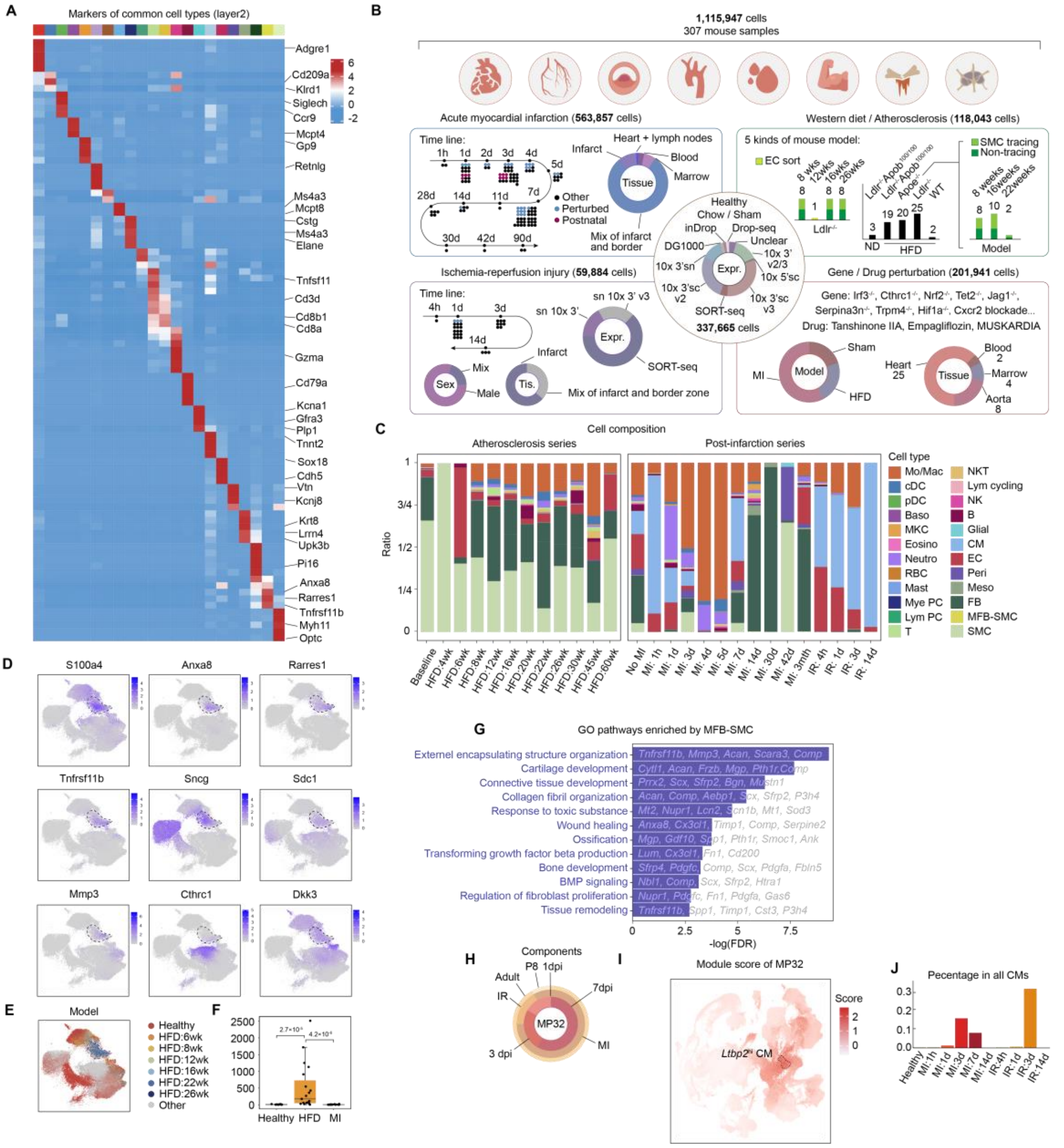

Figure S3 IHDAtlas allows in-depth investigation of single-cell heterogeneity across covariates. (A) Transcriptional markers of cell types annotated by layer3; (B) Meta-information on samples included by ssIHDB; (C) Cell type compositions of each model; (D) Transcriptional markers of MFB-SMC; (E) Embedding of SMCs, FBs, and mesothelial cells and the model distribution of them indicated highlighted the later occurrence of MFB-SMCs in HFD model; (F) Cell counts of MFB-SMCs in the healthy, HFD, and MI model. Each dot represents a sample; (G) Top enriched GO pathways of MFB-SMCs. (H) Distribution of MP32 occurrence across sample covariates; (I) Score of MP32 in IHDAtlas; (J) Percentage of *Ltbp2*^{hi} CM in all CMs at time points after AMI or IR injury. FB, fibroblast; Meso, mesothelial cell, SMC, smooth muscle cell; Peri, pericyte; Neu, neutrophil; EC, endothelial cell; cMo, classical monocyte; ncMo, non-

classical monocyte; cDC, classical dendritic cell; pDC, plasmacytoid dendritic cell; Mac, macrophage; GO, gene ontology; MI, myocardial infarction; HFD, high-fat diet; FDR, false discovery rate.

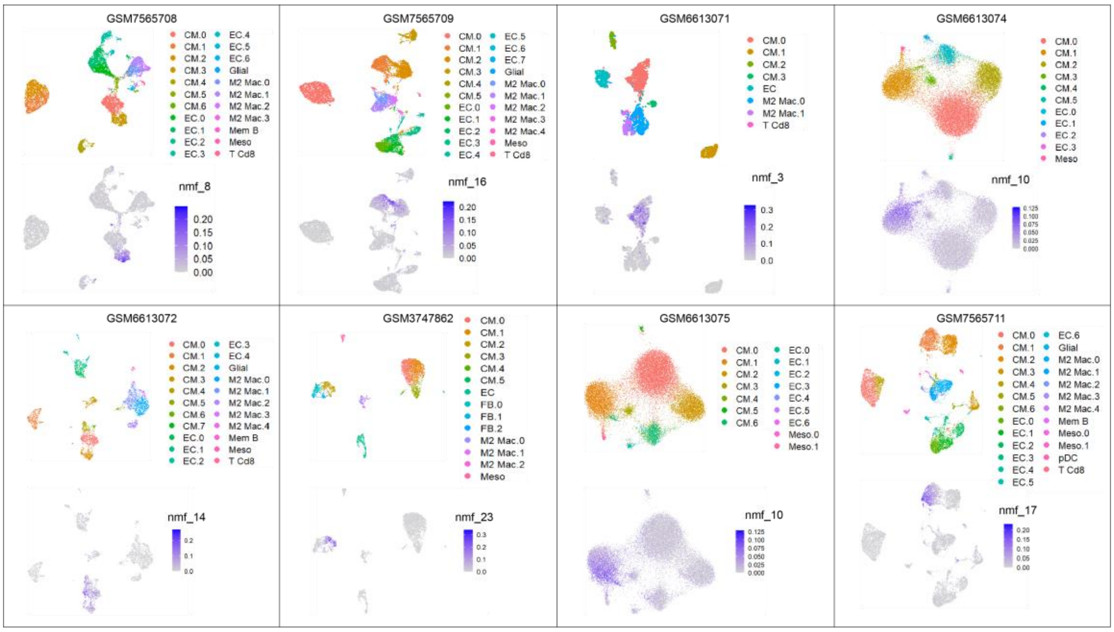

Figure S4 Samples with cardiomyocytes highly expressing MP32. Each subgraph shows the cell type constitution of one of the samples that contribute to MP32 and the scores of the corresponding program that belongs to MP32.

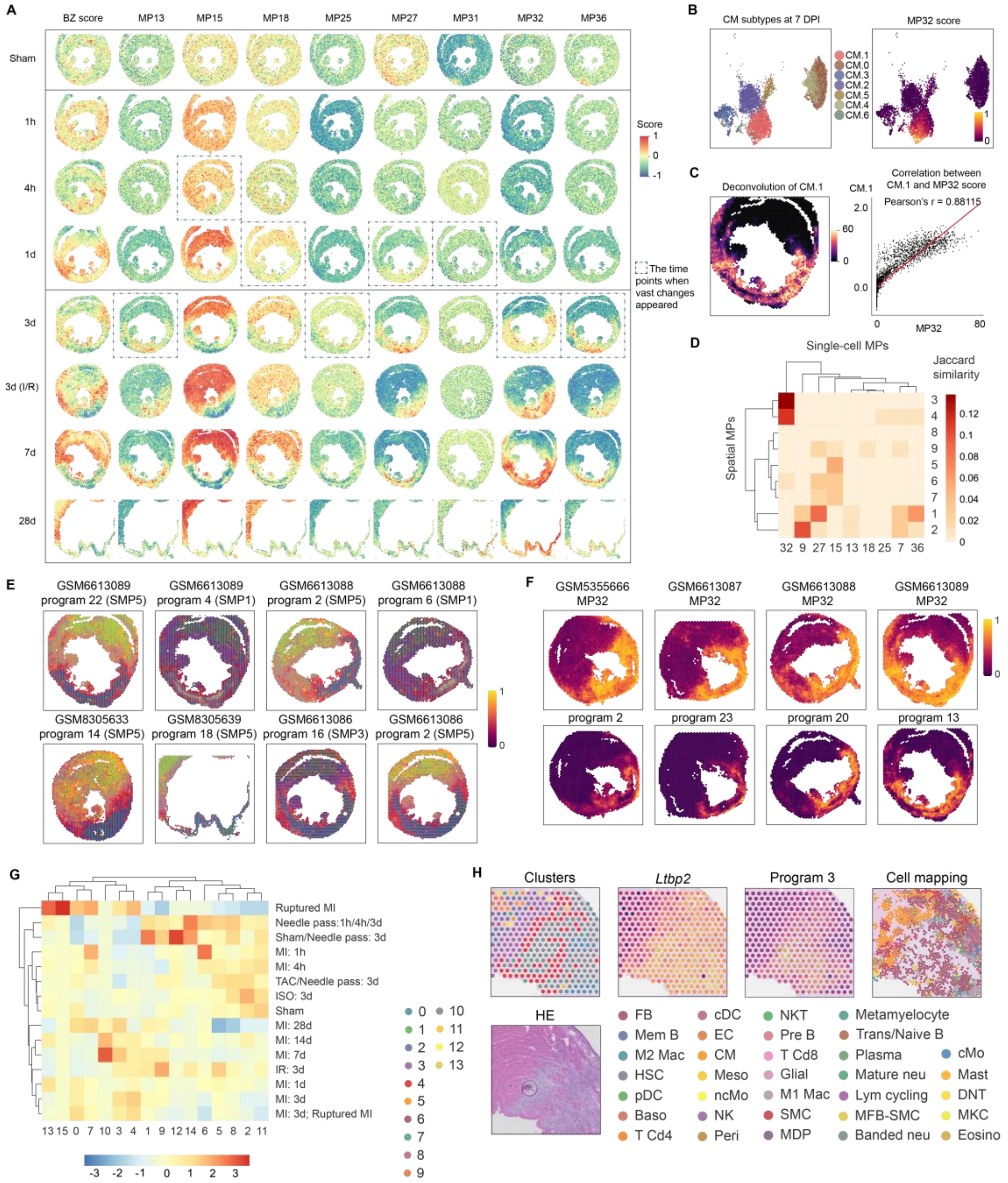

Figure S5 Meta-programs define responsive patterns of infarcted myocardium at single-cell and spatial resolution. (A) Scores of AMI- and IR-related MPs in a series of ST samples; (B) CM subtypes from sample GSM7565711 (left) and MP32 usage of the CMs (right); (C) Deconvolution of *Ltbp2*^{hi} CM from to 7 DPI ST data by cell2location (left) and the correlation between CM subtype 1 distribution and MP32 usage (right). (D) Correlation of single-cell MPs and spatial MPs; (E) Consistent zonation of phenotypes demonstrated by stacked graphs of spatial NRP usage and spatial domains; (F) Comparison of MP32 usage and SMP usage in the same slices; (G) Model composition of spot clusters; (H) Spatial visualization module of ssIHDB web portal. Graphs were generated by ssIHDB.

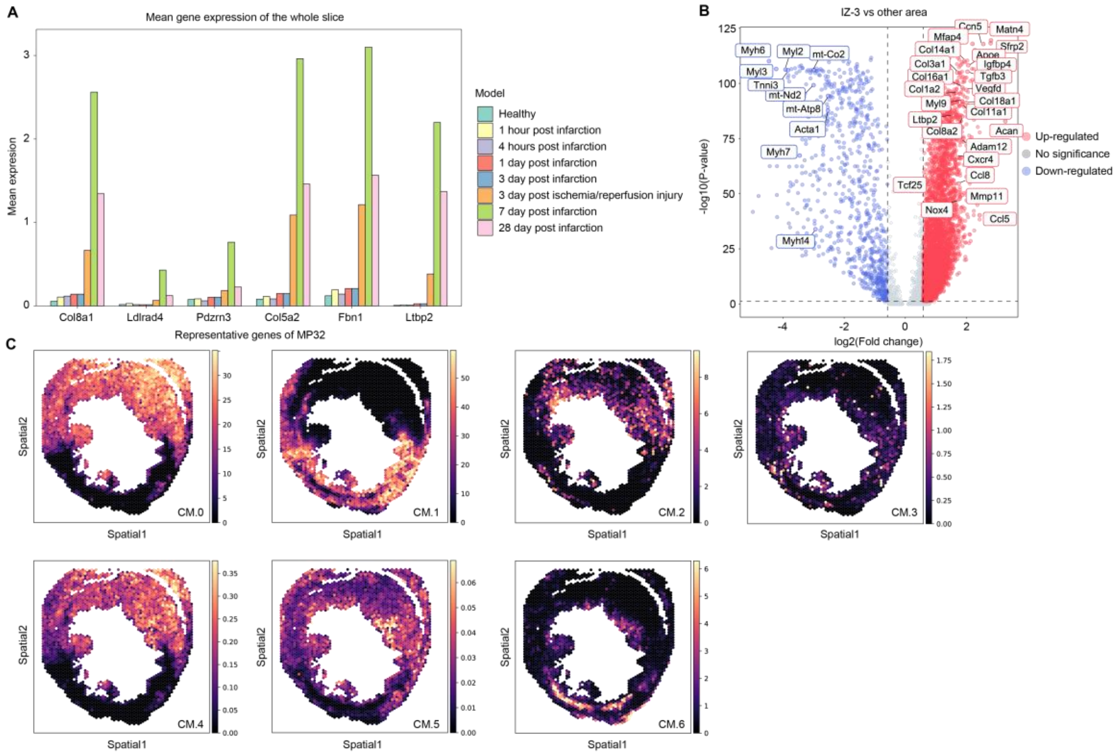

Figure S6 MP32 was spatio-temporally regulated after myocardial infarction. (A) Mean expression of genes representative of MP32 in ST datasets post-infarction; (B) Differentially expressed genes of IZ-C compared to other regions at 7 DPI; (C) Estimated abundance of CM subtypes from GSM7565711 at all spots from ST data at 7 DPI.

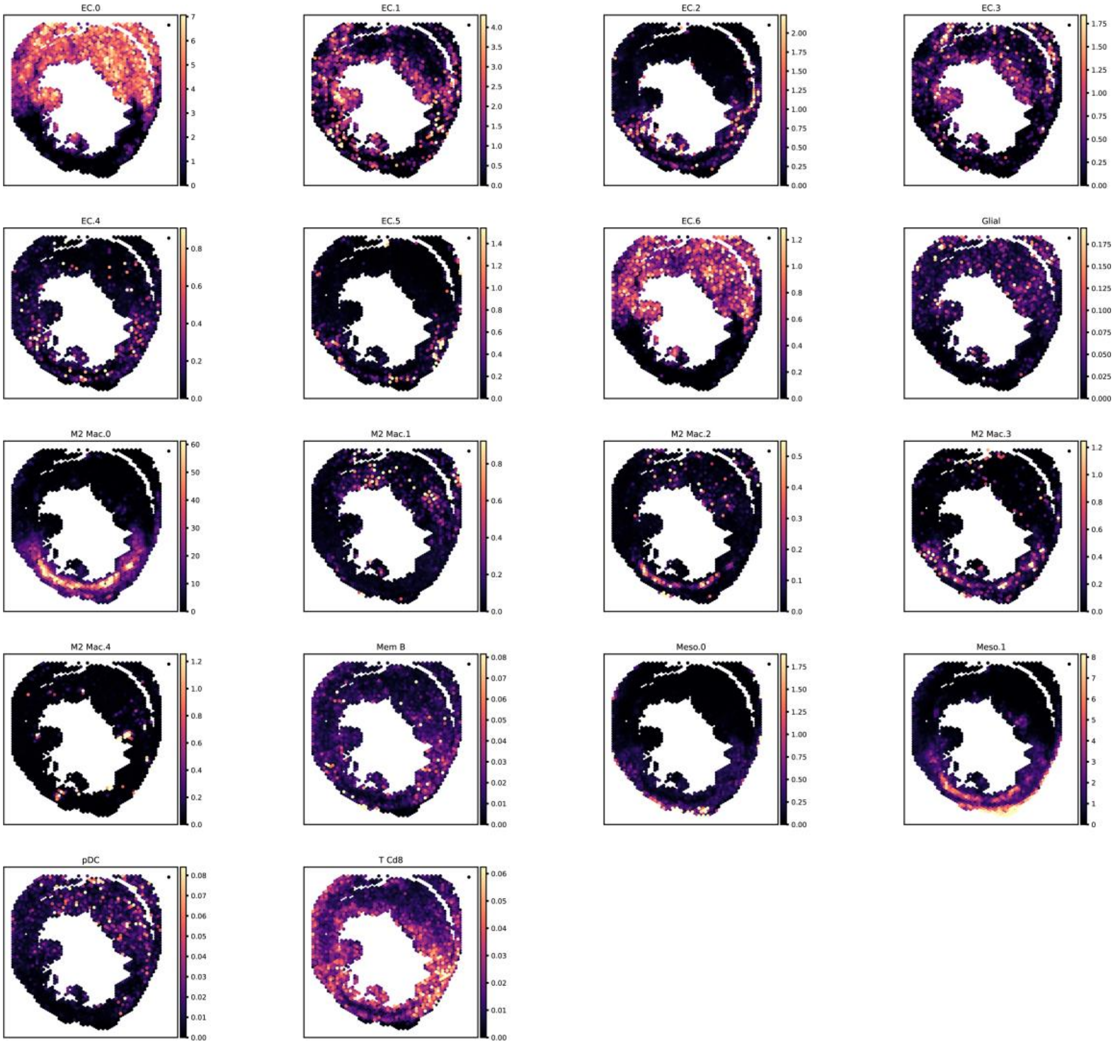

Figure S7 Spatial deconvolution of non-CM cell subtypes. Each subgraph shows the estimated abundance of a cell subtype at all spots from ST data at 7 DPI.

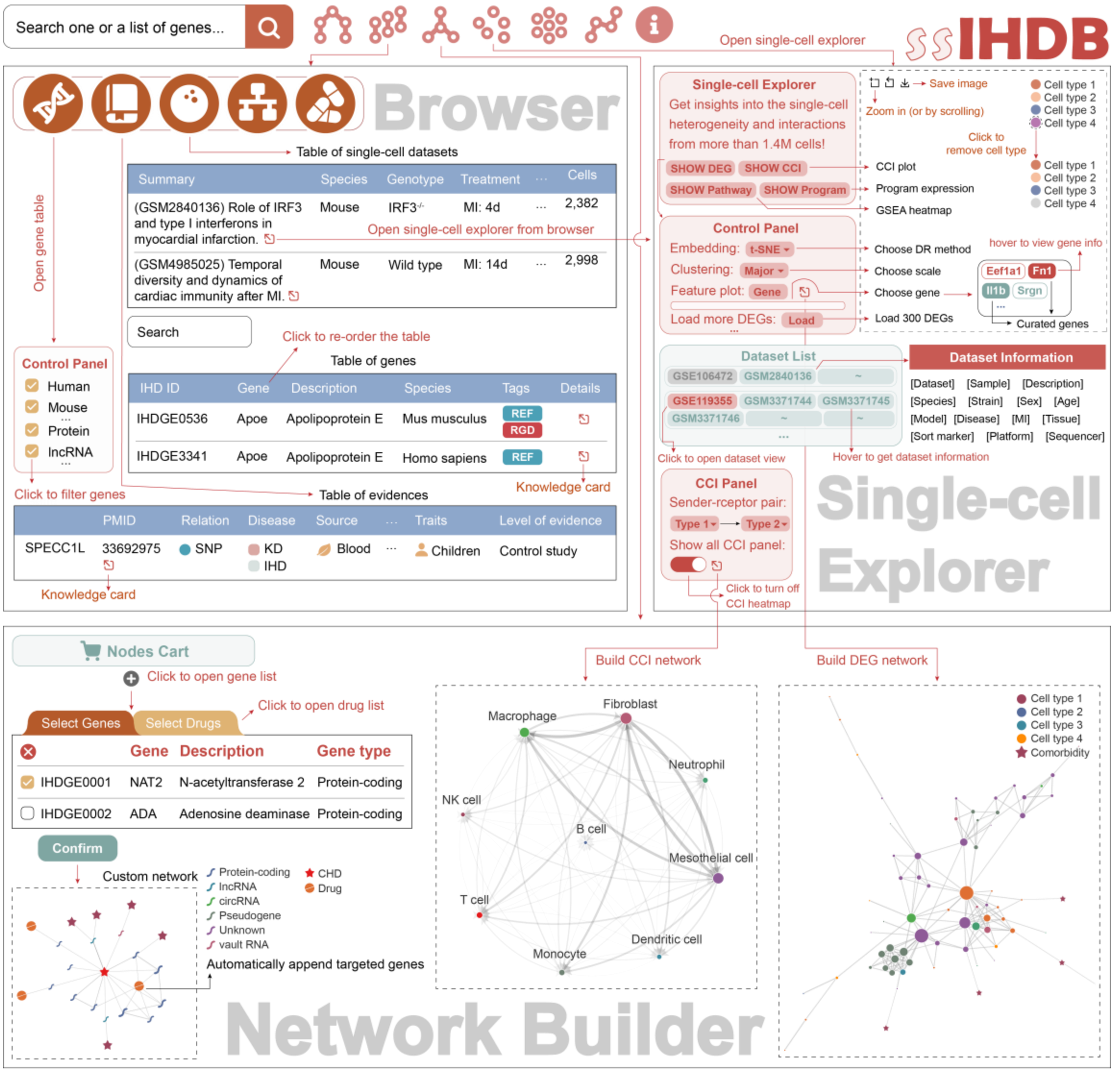

Figure S8 Instructions for using the ssIHDB website. Red arrows indicate click. Black arrows are the explanation. This tutorial shows how to view basic information on genes, and scRNA-seq datasets related to IHD, and how to visualize cell features in the single-cell explorer and build networks using the network builder. This graph also shows how data from different parts can be integrated with great freedom.

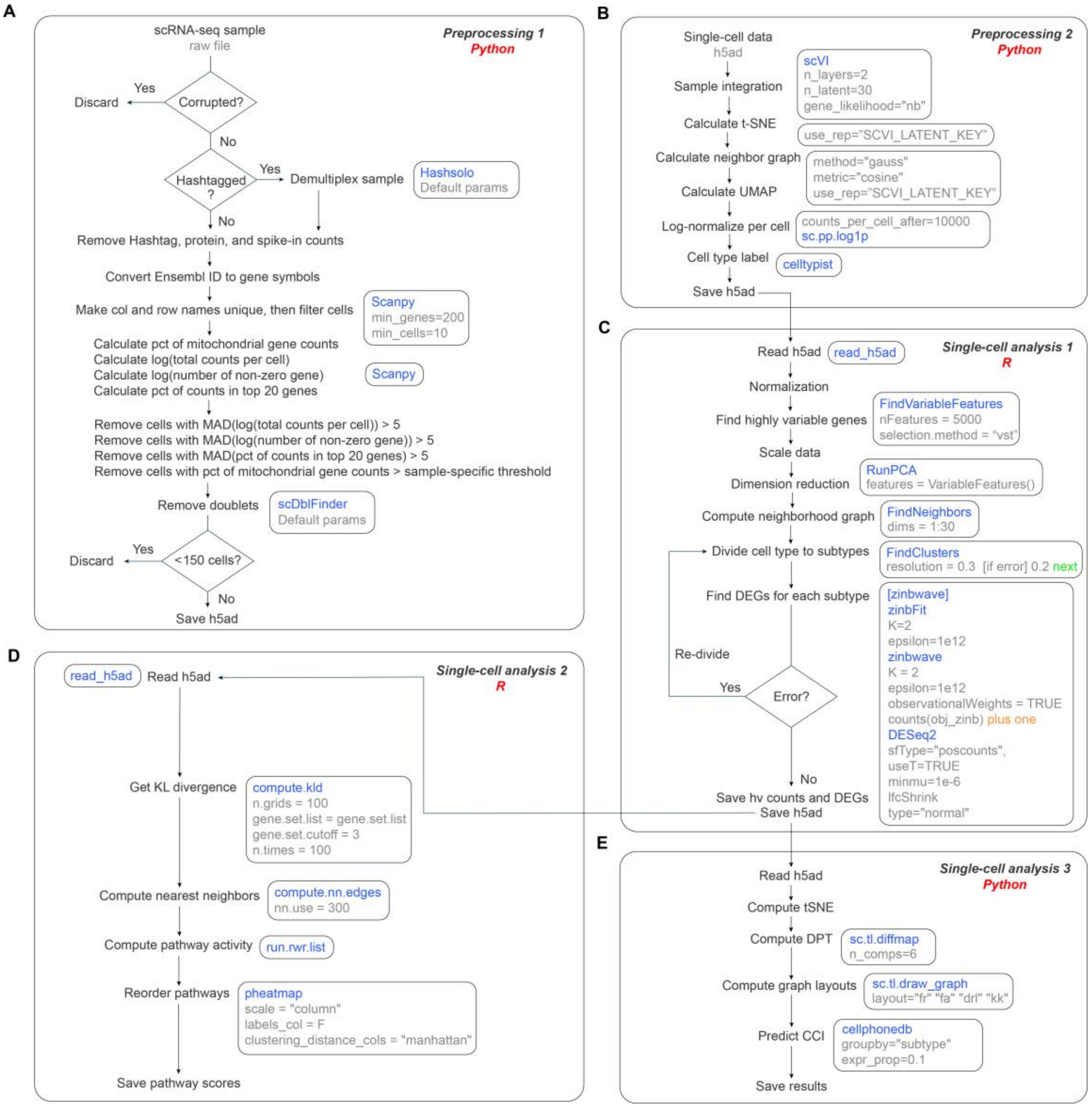

Figure S9 ScRNA-seq data processing workflow for ssIHDB. (A) Preprocessing step 1, quality control of data samples; (B) Preprocessing step 2, basic data transformation, dimension reduction, and cell label prediction; (C) Analysis step 1, find highly variable genes by DESeq2 with ZinbWave recipe and define cell subtypes; (D) Analysis step 2, compute subtype-level pathway activeness by GSDensity; (E) Analysis step 3, predict subtype-level CCI and compute other layouts.

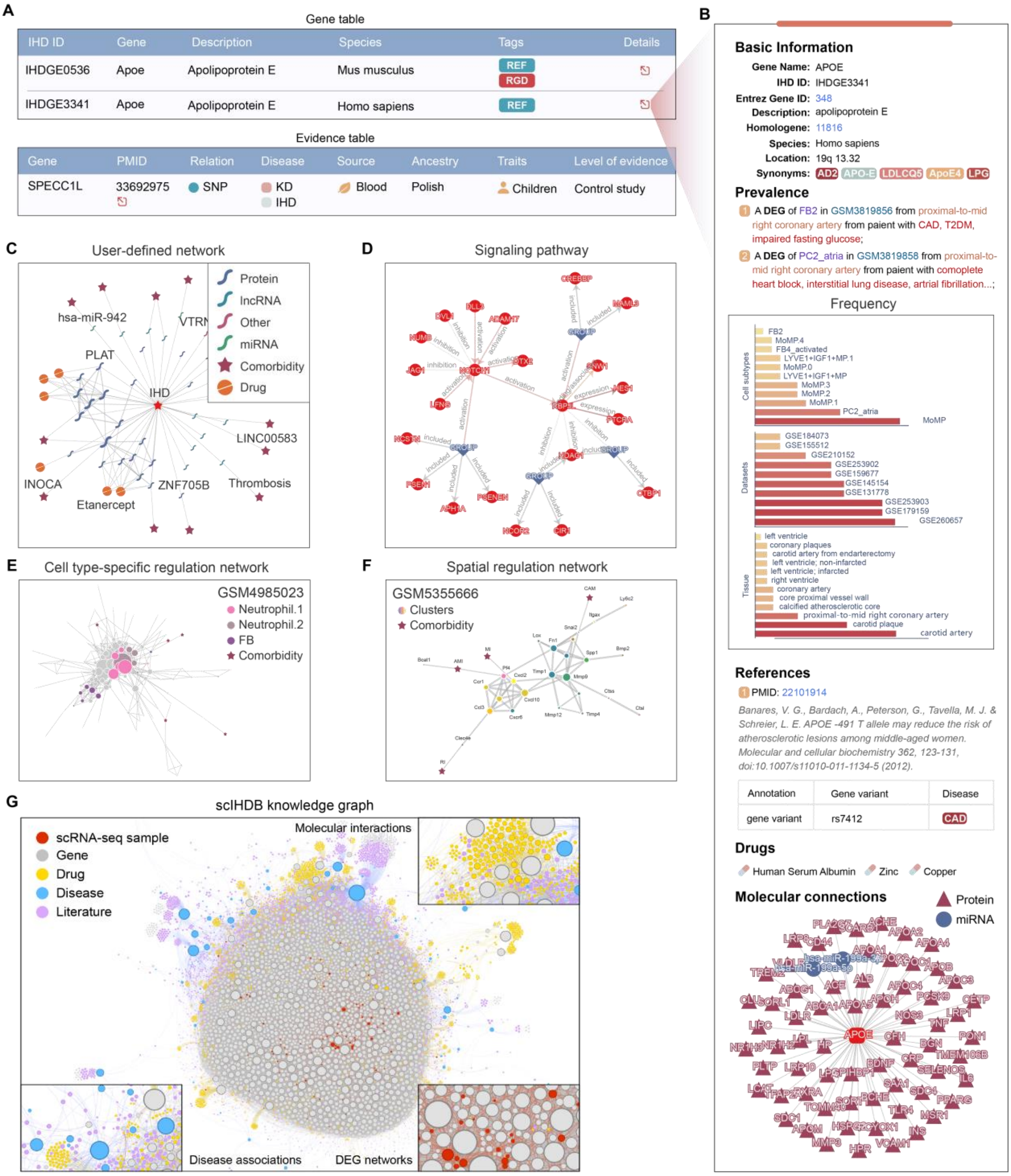

Figure S10 SsIHDB browser and network builder allows multi-level network analysis. (A) SsIHDB browser lists IHD-related genes, supporting evidence, single-cell samples, pathways, and drugs, each correspond to a node in the knowledge graph; (B) Each node has a knowledge card. Here, a gene card shows the basic information, prevalence, evidence, drugs, and molecular interactions associated to the gene; (C) A user-defined network constructed by the network builder. The node size was positively related to its degree; (D) A graph representation of Notch signaling pathway in pathway knowledge card. (E) A DE network with cell subtype-specific genes highlighted; (F) A spatial DE

network, each node represents a DE gene of a specific spot cluster; (G) The whole knowledge graph involves genes, evidence, drugs, comorbidities, and single-cell DE genes. Networks were generated and exported from ssIHDB website.

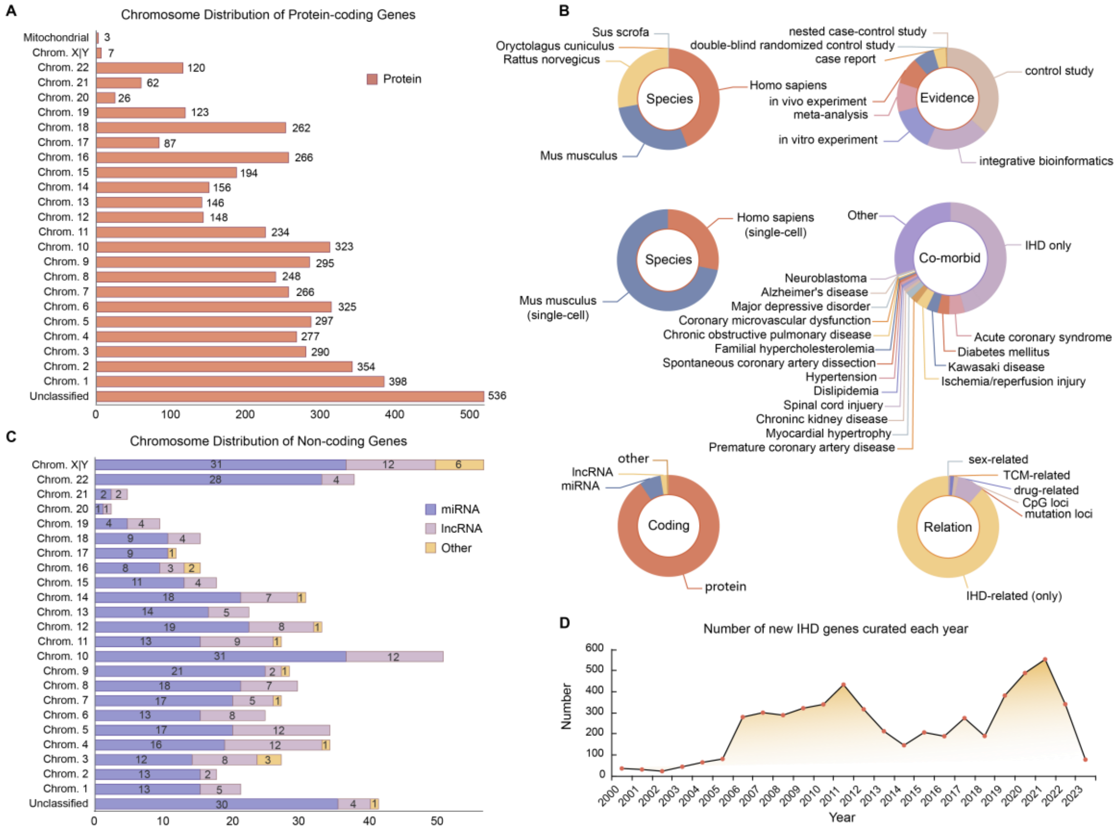

Figure S11 Statistics of ssIHDB. (A) Distribution of the curated 5444 protein-coding genes on chromosomes; (B) Top left, proportion of genes for each species. Top right, proportion of evidence of different confidence levels. Middle left, proportion of scRNA-seq datasets from each species. Middle right, ratio of IHD comorbidities related to curated genes. Bottom left, proportion of genes belonging to each molecule category. Bottom right, proportion of each category of gene-disease associations; (C) Distribution of non-coding genes on chromosomes; (D) Number of reference articles that found new IHD-related genes each year. TCM, traditional Chinese medicine.

4. Supporting manual of ssIHDB

Welcome to ssIHDB! SsIHDB is a single-cell and spatio-temporally resolved database for ischemic heart disease (IHD). This brief user guide will help you quickly familiarize yourself with the core functions of ssIHDB. Do not like reading manuals? No problem! You can also quickly and accurately understand the website's features by referring to the abundant tooltips available throughout ssIHDB. Now, let's explore the 7 major modules of ssIHDB together.

3.1. Home

The homepage provides a brief introduction to the main modules of ssIHDB and their meta-information, including literature, network tools, single-cell and spatial exploration. You can hover your mouse over each icon to view basic descriptions of each module.

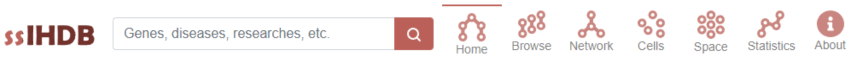

3.2. The "Browse" module

The Browse module consists of five fundamental literature resources in ssIHDB: genes, literature evidence, single-cell datasets, pathways, and drugs. Users can reorder columns by clicking the headers and access detailed information by clicking the corresponding icons.

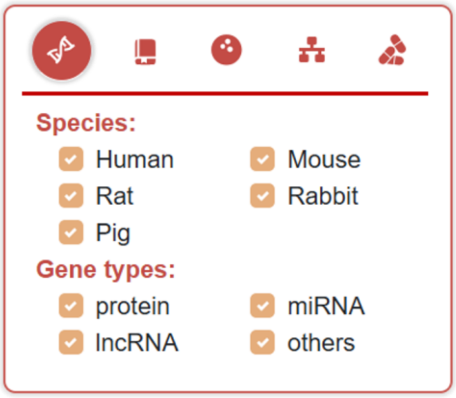

For genes, ssIHDB provides basic information in the table. Details such as gene locus, synonyms, referenced articles, gene-gene interactions, and implicated drugs can be accessed by clicking the "details" button. Users can also filter genes by specific categories or species and search for a particular gene using the search box.

IHD ID	Gene	Description	Species	Tags	Details
IHDGE0001	NAT2	N-acetyltransferase 2	Homo sapiens	RGD REF	
IHDGE0002	ADA	adenosine deaminase	Homo sapiens	RGD REF	
IHDGE0003	CDH2	cadherin 2	Homo sapiens	REF	
IHDGE0004	AKT3	AKT serine/threonine kinase 3	Homo sapiens	REF	
IHDGE0005	SCN9A	sodium voltage-gated channel alpha subunit 9	Oryctolagus cuniculus	REF	
IHDGE0006	Ccdc92	coiled-coil domain containing 92	Rattus norvegicus	RGD	
IHDGE0007	Mirt2	myocardial infraction associated transcript 2	Mus musculus	REF	
IHDGE0008	Gm2023	predicted gene 2023	Mus musculus	REF	

Search Showing 1 to 8 of 6,035 entries Previous 1 2 3 4 5 ... 755 Next

For references, the table details how each gene is related to IHD. The "relation" column indicates the type of association (*e.g.*, gene mutation, DNA methylation, drug effect, gender effect). The "disease" column shows which IHD comorbidities are linked to the gene. The "source" column indicates the tissue or cell line used to establish the association. The "ancestry" column shows the ethnicity of study participants. The "trait" column displays other participant characteristics. The "level of evidence" column indicates the strength of the evidence from the study.

Gene	PMID	Relation	Disease	Source	Ancestry	Traits	Level of evidence
GAS5	35235104	SNP	IHD	NA	Chinese	age<60	control study
ESR1	35235104	SNP	IHD	NA	Chinese	age<60	control study
BPI	34387532	related	AMI IHD	plasma	Chinese	Anhui province	cohort study
NRG4	30393265	related	IHD	plasma	Chinese	Beijing	control study
MIR4497	31443660	related	UA IHD	PBMC	Chinese	Beijing	control study
MIR320B1	33536383	related	IHD	NA	Chinese	Beijing	control study
MIR320B2	33536383	related	IHD	NA	Chinese	Beijing	control study

Search Showing 1 to 7 of 6,773 entries Previous 1 2 3 4 5 ... 968 Next

For single-cell datasets, the table provides basic information for each sample, including species, genotype or ethnicity, model treatment, tissue source, cell markers used for sorting, and cell counts.

Summary	Species	Genotype	Treatment	Source	Sorted	Cells
(ERS2921927) Single-cell expression profiling reveals dynamic flux of cardiac stromal, vascular and immune cells in health and injury	mouse	Pdgfra(GFP/+)	MI:3d	ventricle	CD31-; GFP+	3306
(ERS2921928) Single-cell expression profiling reveals dynamic flux of cardiac stromal, vascular and immune cells in health and injury	mouse	Pdgfra(GFP/+)	sham:3d	ventricle	CD31-; GFP+	2181
(ERS2921929) Single-cell expression profiling reveals dynamic flux of cardiac stromal, vascular and immune cells in health and injury	mouse	Pdgfra(GFP/+)	MI:7d	ventricle	CD31-; GFP+	5312
(ERS2921930) Single-cell expression profiling reveals dynamic flux of cardiac stromal, vascular and immune cells in health and injury	mouse	Pdgfra(GFP/+)	sham:7d	ventricle	CD31-; GFP+	5048
(ERS2921931) Single-cell expression profiling reveals dynamic flux of cardiac	mouse	Pdgfra(GFP/+)	MI:3d	ventricle	-	3639

For pathways and drugs, the table also provides essential information for each item. Clicking the "details" button in the pathway table opens a dynamic graph showing all pathway components and their functional relationships.

IHD ID	KEGG ID	Pathway Name	Species	Details
IHDPATH001	hsa04010	MAPK signaling pathway	Homo sapiens	
IHDPATH002	hsa04012	ErbB signaling pathway	Homo sapiens	
IHDPATH003	hsa04020	Calcium signaling pathway	Homo sapiens	
IHDPATH004	hsa04064	NF-kappa B signaling pathway	Homo sapiens	
IHDPATH005	hsa04066	HIF-1 signaling pathway	Homo sapiens	
IHDPATH006	hsa04070	Phosphatidylinositol signaling system	Homo sapiens	
IHDPATH007	hsa04115	p53 signaling pathway	Homo sapiens	
IHDPATH008	hsa04150	mTOR signaling pathway	Homo sapiens	
IHDPATH009	hsa04151	PI3K-Akt signaling pathway	Homo sapiens	
IHDPATH010	hsa04210	Apoptosis	Homo sapiens	
IHDPATH011	hsa04310	Wnt signaling pathway	Homo sapiens	
IHDPATH012	hsa04330	Notch signaling pathway	Homo sapiens	

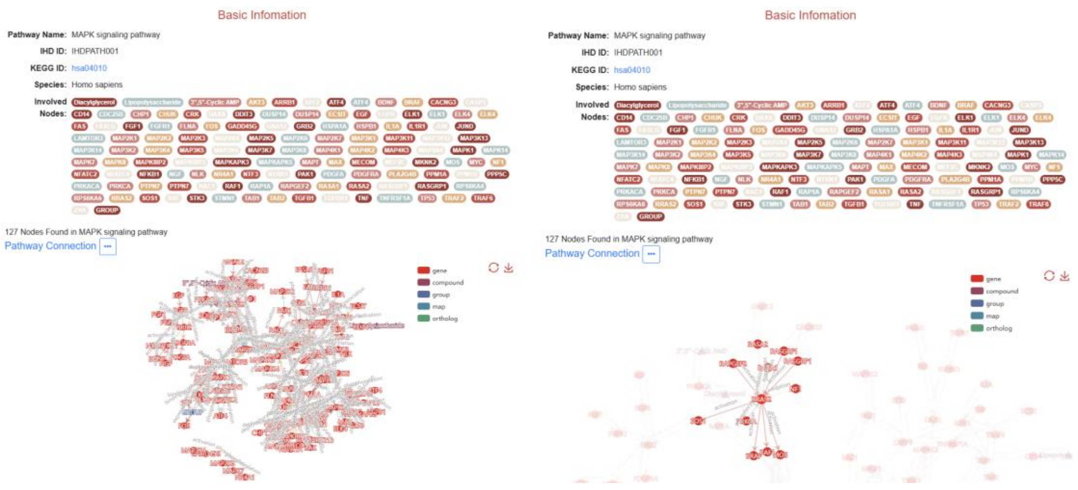

3.3. The "Network" module

The Network module allows users to build custom pharmaceutical networks based on IHD-related genes, drugs, diseases, and single-cell or spatially differentially expressed genes. Genes are classified into protein-coding, lncRNA, miRNA, circRNA, pseudogene, vault RNA, and unknown types. Users can construct networks using either an evidence-driven or data-driven pattern.

For the **evidence-driven pattern**, users should have prior knowledge of IHD and specific target genes or drugs in mind. They can select genes and drugs of interest in the nodes cart and click the confirm button.

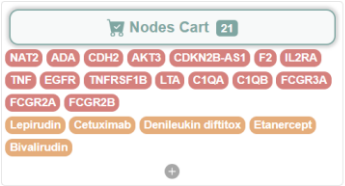

For the **data-driven pattern**, users can utilize differentially expressed genes from the "Cells" or "Space" module, generated through single-cell or spatial analysis across clusters or spatial domains. Due to the high computational demand, results may take a moment to display.

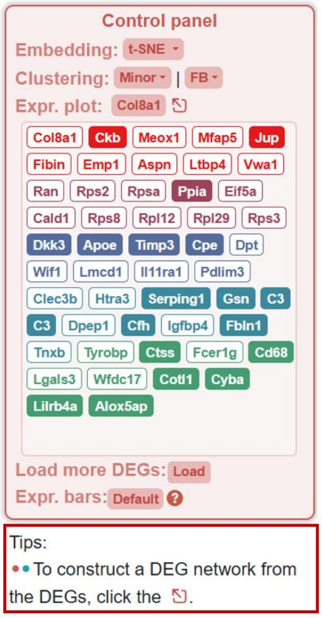

This integrated network visualization module incorporates multiple functional annotation databases, including STRING, LncACTdb3.0, miRTarBase, DrugBank, circBase, etc., along with manually curated molecular interaction annotations. Users can easily access functional annotations for all nodes in the network.

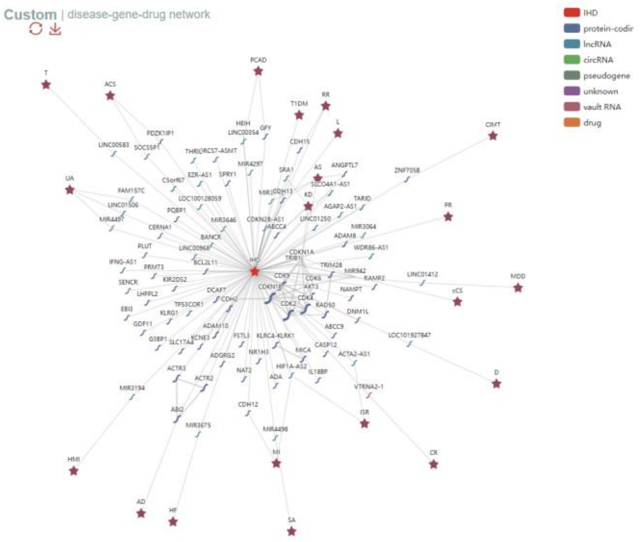

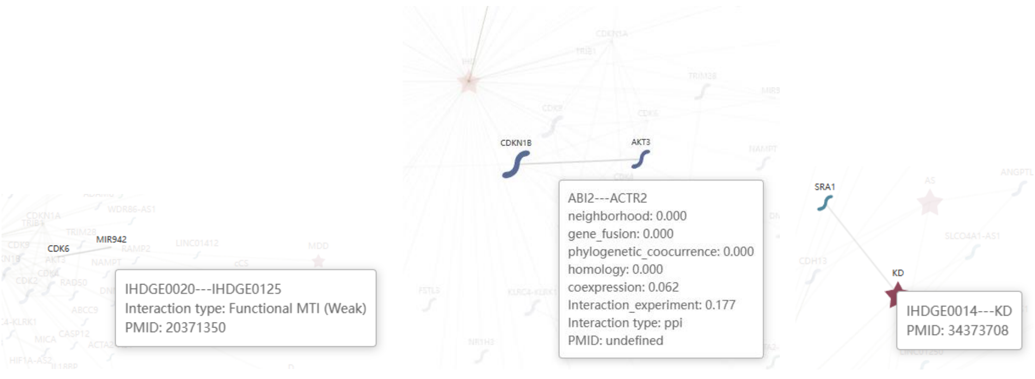

3.4. The "Cells" module

The Cells module is a comprehensive visualization interface that presents single-cell data analysis results collected in the Browse module. Users can switch between different single-cell explorers for various datasets, focusing on **DEG**, **CCI**, **pathway**, and **program**. Visualization modes can be selected from the control panel. Dataset information is displayed at the bottom of the explorer.

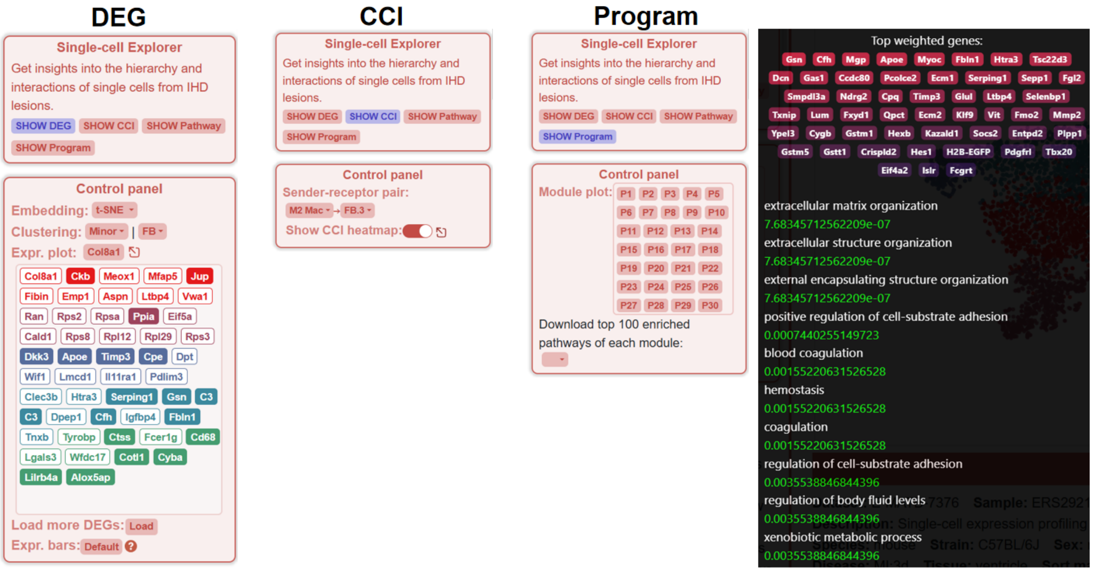

In the DEG explorer, cluster annotations are shown in the top-right corner. Detailed gene expression data can be viewed by hovering over individual cells. When a DEG of interest is selected, a gradient-colored UMAP plot based on gene expression levels will appear.

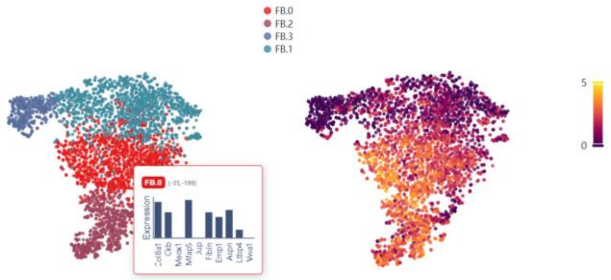

After clicking the "SHOW CCI" button, users can select a sender-receptor cell type pair from the heatmap or the control panel, and a dot plot of ligand-receptor interaction strengths will be displayed.

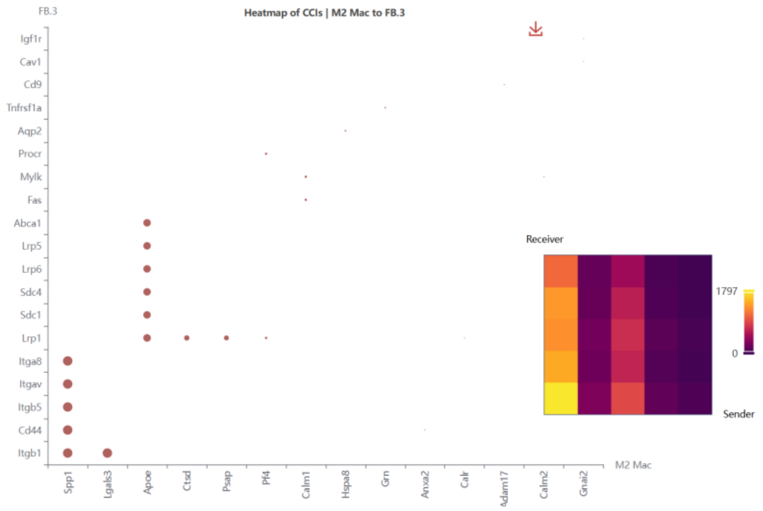

Clicking the "SHOW Pathway" button displays a heatmap of highly variable pathways. Hovering over the heatmap reveals the selected pathway and its average activity.

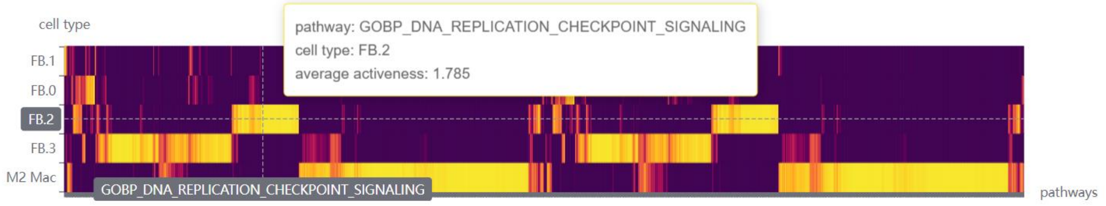

Clicking the "SHOW Program" button displays NMF program scores. All explorers can be zoomed in or out using the mouse for detailed observation.

3.5. The "Space" module

The "Space" module provides an interactive visualization interface for exploring IHD-related spatial transcriptomics data. It includes a basic graph on the left showing spatial spot locations and a differential expression (DE) graph on the right showing DE patterns for up to 300 genes. The DE explorer shares the same structure as in the Cells module. Spatial domains 0–13 were identified from the integrated spot atlas of AMI mouse. Available spot layouts include spatial location and UMAP embedding.

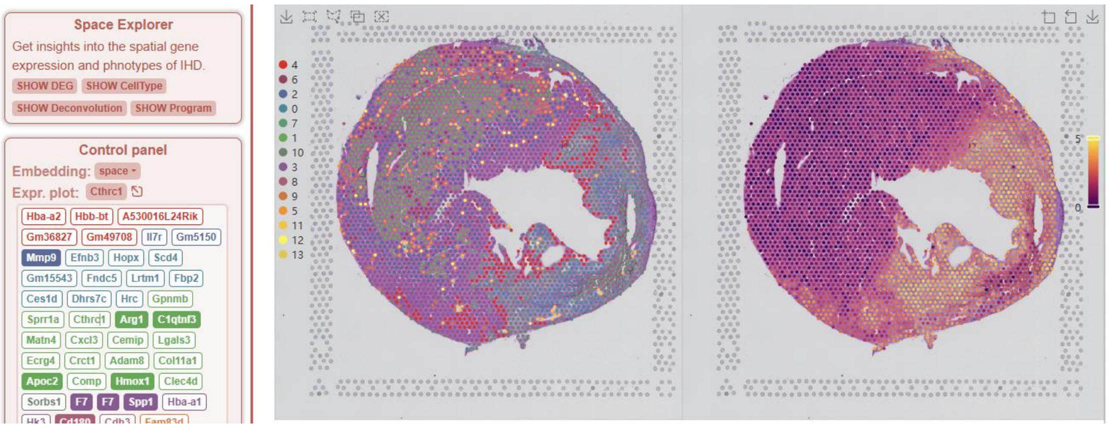

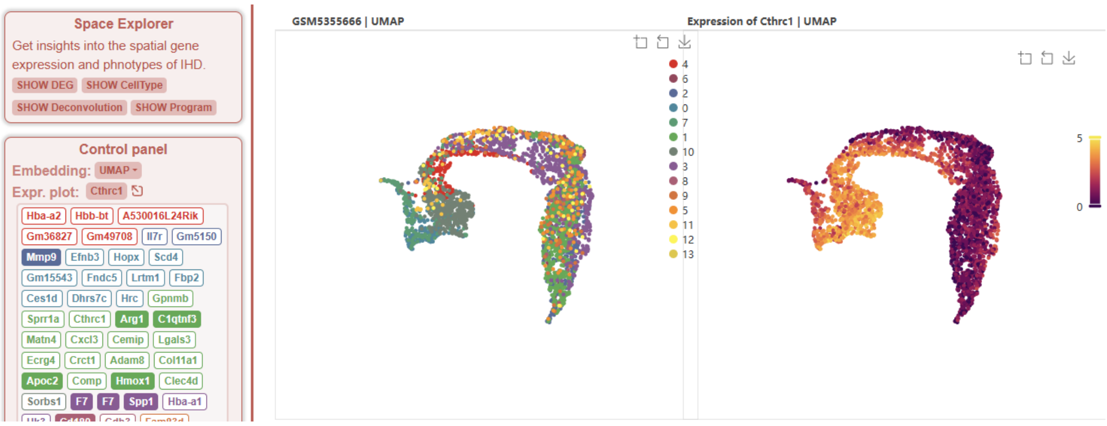

SsIHDB supports online differential expression analysis for user-selected spot groups. Click "Show brush statistics panel" to see a box where selected spots are displayed. Use the "Lasso select" or "Box select" tool to select spots in an enclosed area. Selected spots will appear in the box after releasing the mouse. To select spots from multiple areas, click "Keep Selections" and continue selecting. All selected spots will be displayed.

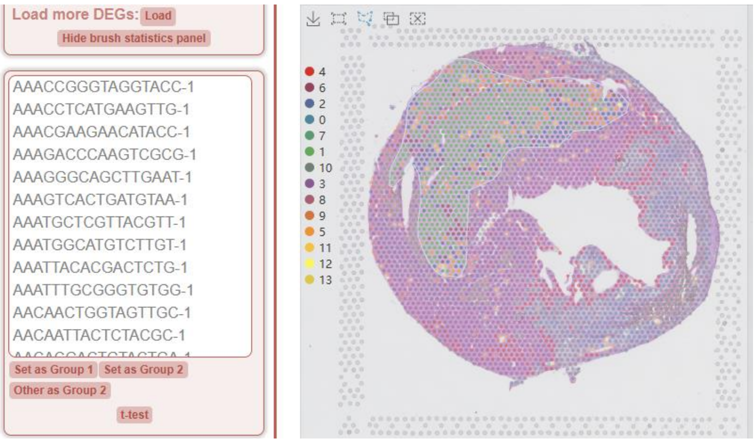

Click "Set as group 1" to assign the selected spots as the functional group.

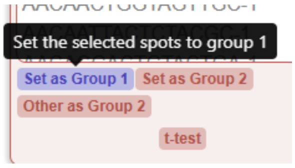

Then, either click "Other as group 2" to set all other spots as the reference group, or click "Clear selection" to remove current selections and choose another group of spots. Click "Set as group 2" to assign them as the reference group.

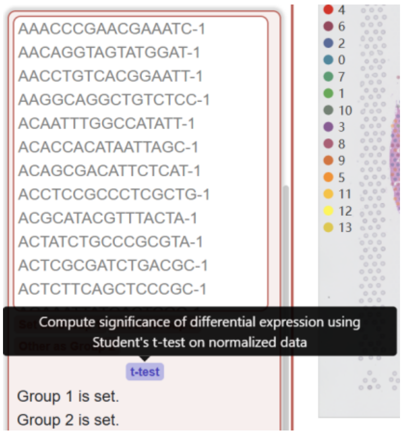

Finally, click the "t-test" button. The expression matrix will be downloaded automatically, and a Student's *t*-test will be performed. Once completed, a CSV file with all results will be downloaded.

gene	mean_1	mean_2	t	p	confidence	confidence	valid	freedom
Ech1	4.812391	3.676039	61.17422	0	1.099929	1.172774	TRUE	2934.394
Acaa2	4.018726	2.894542	55.78174	0	1.084668	1.1637	TRUE	2996.202
Idh2	4.3554	3.335063	54.67562	0	0.983745	1.056928	TRUE	2924.423
Gm15543	3.898495	2.353551	51.95459	0	1.486635	1.603253	TRUE	2650.561
Slc25a4	6.02156	5.094843	51.73953	0	0.891594	0.961839	TRUE	2434.926
Cox7a1	5.666644	4.394417	51.67015	0	1.223945	1.320509	TRUE	2465.298
Lpl	5.120694	4.078784	51.62432	0	1.002336	1.081485	TRUE	2735.486
Acadm	4.049232	2.861266	51.42203	0	1.142667	1.233264	TRUE	2901.975
Hopx	3.045508	1.614786	50.49012	0	1.375162	1.486282	TRUE	3107.622
Tnni3	5.889895	4.589024	49.48196	0	1.249317	1.352423	TRUE	2404.014
Fabp3	6.43677	5.134785	49.45025	0	1.250354	1.353615	TRUE	2369.167
Myl3	6.3684	5.09374	49.42948	0	1.224091	1.325228	TRUE	2357.006
Atp5g3	5.034605	4.328528	49.20234	0	0.677938	0.734215	TRUE	2834.572
Atp2a2	6.006324	4.917145	49.08585	0	1.045667	1.13269	TRUE	2483.959
Fhl2	4.742531	3.673398	49.00997	0	1.026359	1.111908	TRUE	2790.126
Atp5md	5.384712	4.770597	48.95795	0	0.589519	0.638712	TRUE	2735.83
Ndufv3	4.634783	3.958726	48.91136	0	0.648955	0.703159	TRUE	2919.135
Uqcrb	4.947132	4.195949	48.88323	0	0.721052	0.781315	TRUE	2848.095
Cycs	4.87984	3.9226	48.52853	0	0.918561	0.99592	TRUE	2527.86

Clicking "SHOW Celltype" displays the spatial distribution of CellTrek prediction scores for the selected cell type.

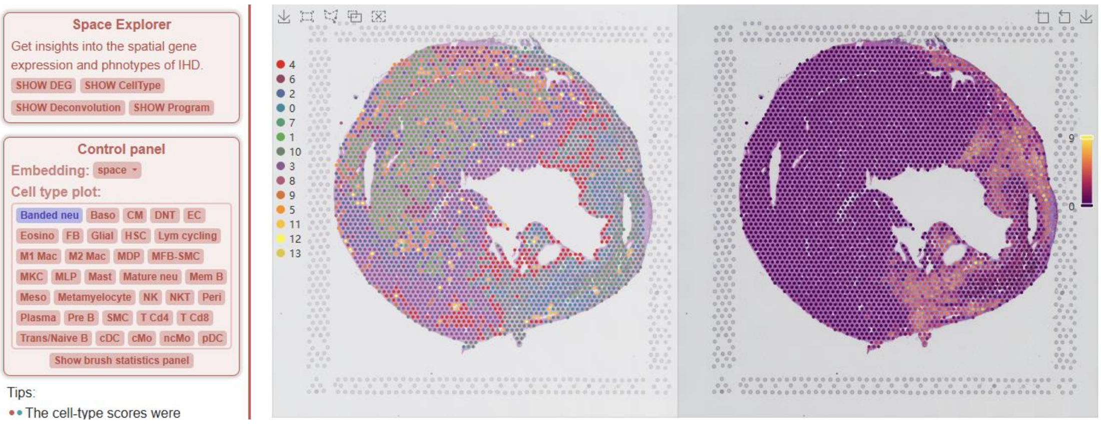

Clicking "SHOW Deconvolution" shows the predicted spatial locations of cells from IHDAtlas *via* cell2location.

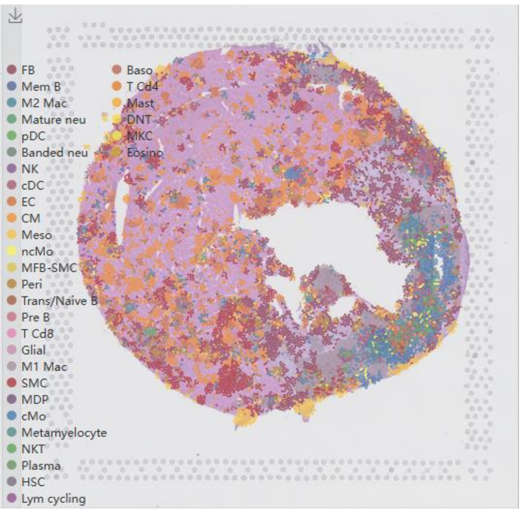

Clicking "SHOW Program" displays NMF program scores for each spot, similar to the Cells module.

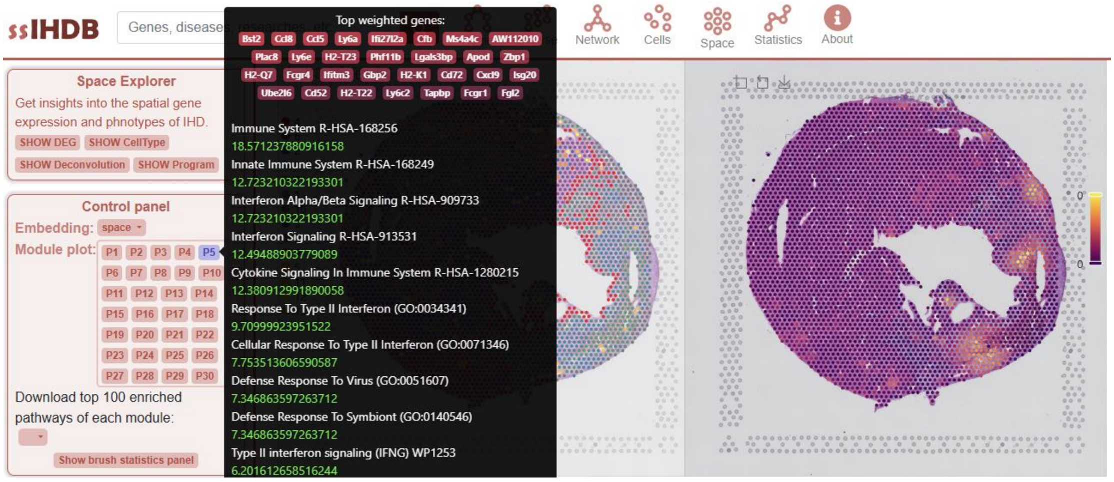

3.6. The "Statistics" module

The Statistics module provides comprehensive statistical information across multiple dimensions for genes, networks, pathways, and meta-programs.

3.7. About

The About module offers a comprehensive introduction to ssIHDB, including content organization, usage guidelines, detailed documentation of methods and download procedures, author information, related publications, and an appendix listing abbreviations and full names of IHD-related comorbidities. For any inquiries, please contact the author at woloorn@zju.edu.cn.

3.8. A step-by-step example: Screening and discovery of potential drug target genes using the online analysis and network pharmacology module on ssIHDB website

[Step 1 (Optional): Select a sample of interest] There are many datasets of different modeling conditions and sequencing platforms. Select one from the dataset panel on the "Space" page and enter the detail page of this sample. For instance, we chose GSM6613087.

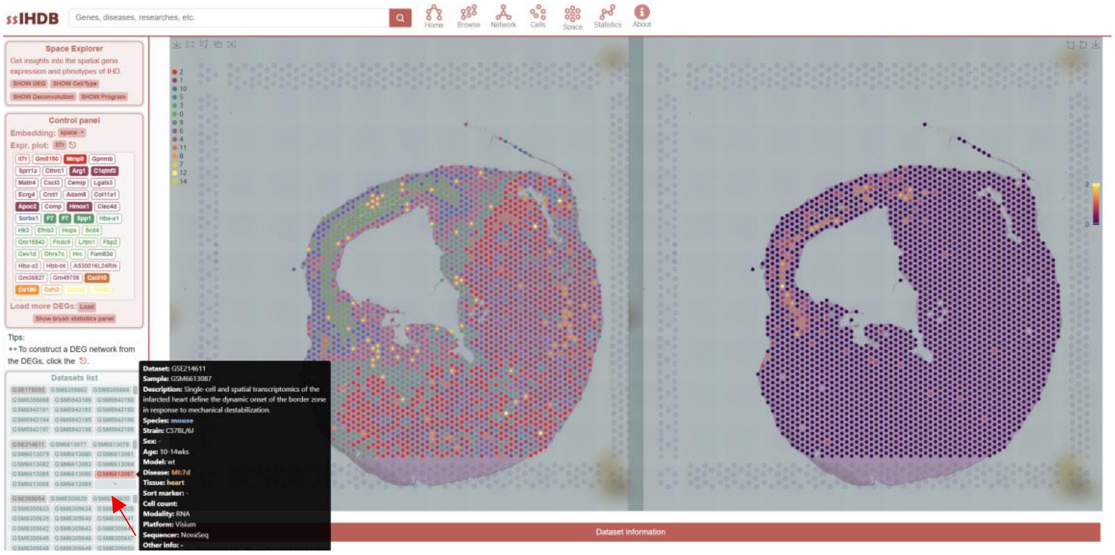

[Step 2 (Optional): Obtain a gene list of interest] Open the statistics panel on the "Space" page, then click the brush button on the upper left of the basic view. You can also turn off some spot clusters by clicking the legends on the upper left to avoid selecting unwanted spots before using the brush.

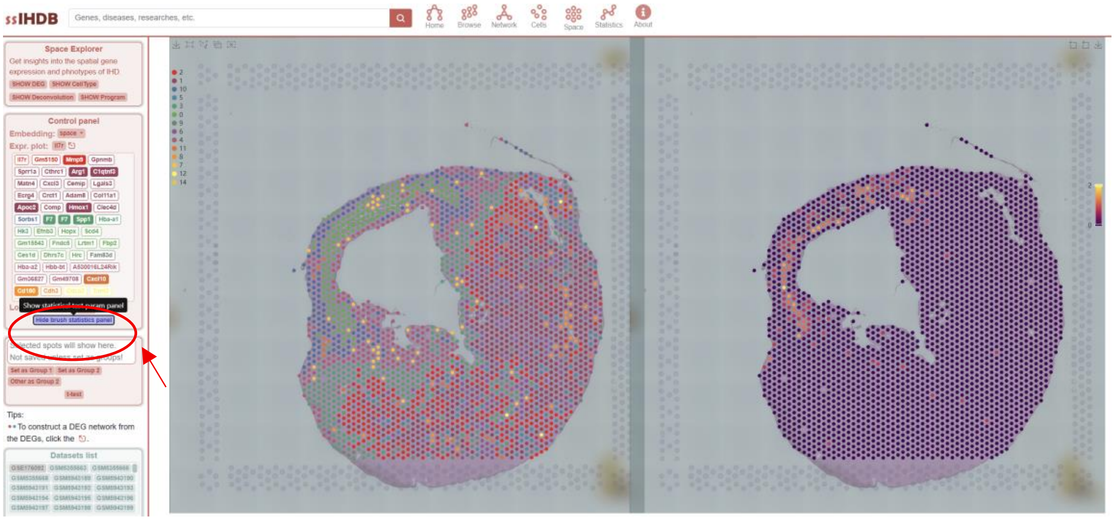

Then, use the brush to create a selection area on the basic view. After every selection (once your left mouse button or touchpad is released), the box in the statistics panel will show the spots that were selected to help you determine if the spots of interest were selected. If the spots of interest are in discrete regions, please click the "keep selection"

button right to the “Lasso select” button first!

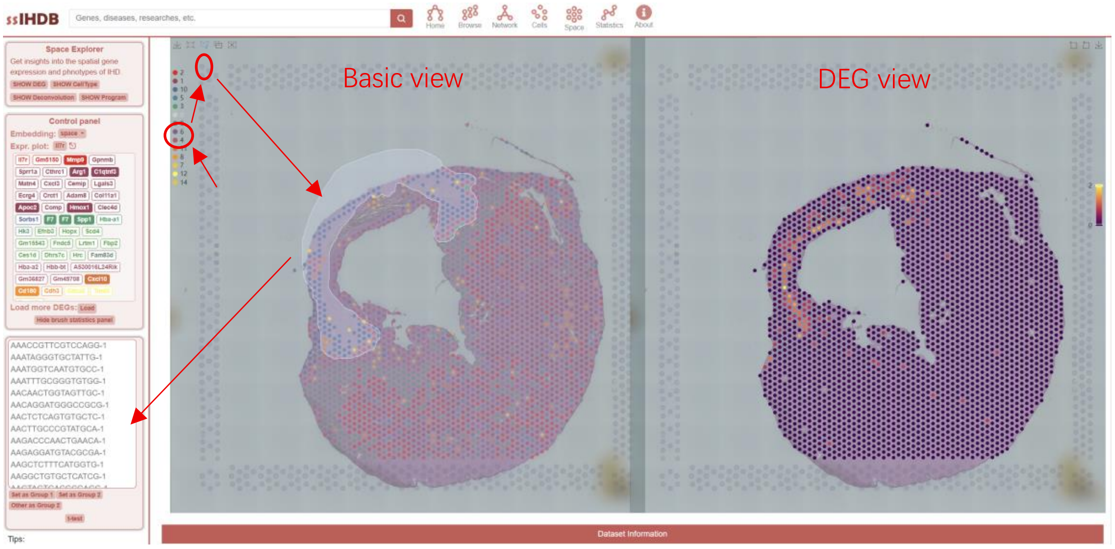

You should click the “Set as Group 1” button to save the selected spots as the group 1 to be compared in t-test. After that you can either click the “Other as Group 2” button or the “Clear Selections” button right to the “Keep Selections” button. The former will set all other spots as the second group to be compared and the latter will allow users to select a second group of spots. In the latter case, you should click the “Set as Group 2” button to save these spots.

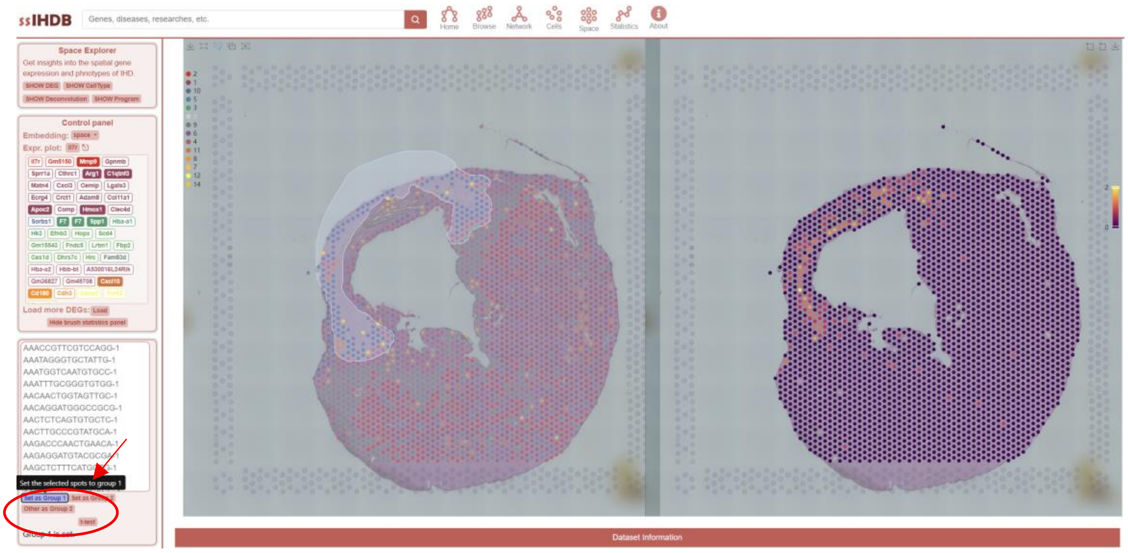

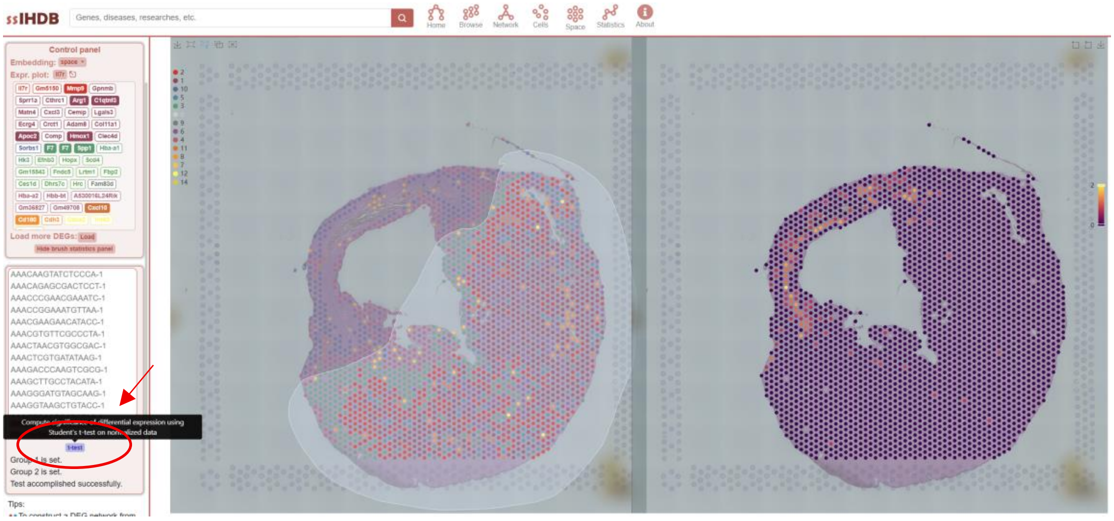

Once group 1 and 2 are both determined, you can click the "t-test" button to start the analysis. The dataset will be automatically downloaded from the database and t-test will be performed on these selected spots. Once the test was completed, the website will return a "t_test_result.csv" which contains p-values of all genes.

=IMLOG2(B2) - IMLOG2(C2)

gene	mean_1	mean_2	t	p	confidence	confidence	valid	freedom	log2FC
Csrp2	4.168341	0.978829	70.56403	0	3.100791	3.278232	TRUE	826.302	2.090344
Col5a1	4.894579	1.771443	65.16277	0	3.029089	3.217183	TRUE	1041.98	1.46626
Col5a2	5.125398	2.009771	64.77431	0	3.021256	3.209998	TRUE	1170.916	1.350633
Fn1	6.395485	2.618167	84.12746	0	3.689235	3.8654	TRUE	1331.151	1.288496
Fbn1	4.882899	2.205125	61.56561	0	2.592447	2.763099	TRUE	1322.329	1.146877
Postn	6.41988	3.19818	69.61224	0	3.130917	3.312482	TRUE	1481.788	1.005295
Cd63	4.482633	2.295078	58.40874	0	2.114085	2.261025	TRUE	1380.507	0.965803
Col1a2	6.91487	3.574555	82.36045	0	3.260751	3.419878	TRUE	1323.42	0.951939
Col1a1	6.960044	3.6047	83.09957	0	3.276127	3.43456	TRUE	1230.624	0.949217
Bgn	6.198502	3.292438	75.89153	0	2.830954	2.981174	TRUE	1559.907	0.912763
Col3a1	7.276857	4.492758	80.96962	0	2.716647	2.851551	TRUE	1366.14	0.695714
Vim	5.379849	3.330016	62.30928	0	1.985284	2.114383	TRUE	1100.666	0.692037
Sparc	6.872458	4.277408	83.70727	0	2.534225	2.655874	TRUE	1173.722	0.684089
Rps12	5.652825	3.810627	69.01552	0	1.789796	1.8946	TRUE	739.711	0.568944
Rps20	5.671569	3.943246	70.43198	0	1.680146	1.776499	TRUE	715.9337	0.524364
Rps16	5.270777	3.734293	63.6844	0	1.489139	1.583828	TRUE	992.7933	0.49718
Rpl18a	5.382303	4.046917	66.0671	0	1.295714	1.375059	TRUE	847.5066	0.4114
Rpl41	5.982799	4.550473	73.44857	5.75E-304	1.394028	1.470624	TRUE	606.2244	0.394804
Rps23	5.378858	3.957097	64.18107	3.78E-295	1.378268	1.465255	TRUE	698.6225	0.442858
Rpl17	5.290708	3.730867	59.25417	8.30E-295	1.508167	1.611514	TRUE	799.8855	0.50395
Ltbp2	4.297128	0.877314	64.3949	5.80E-291	3.31554	3.524088	TRUE	677.3561	2.292208
Rpl4	4.972021	3.330842	53.91364	6.60E-286	1.581437	1.700922	TRUE	912.4548	0.577946
Ccn2	4.633546	1.8012	55.11142	7.22E-285	2.731476	2.933216	TRUE	862.7535	1.363158
Tmsb4x	6.367856	4.419701	71.96469	6.01E-282	1.89498	2.00133	TRUE	549.6936	0.526859
Rpl23	5.456524	4.134832	60.66523	9.91E-282	1.278917	1.364466	TRUE	703.6177	0.400153
Rpl26	5.264307	3.923942	56.48145	5.78E-279	1.293781	1.386949	TRUE	786.2893	0.42394
S100a6	4.647277	2.454008	52.02354	1.09E-278	2.110531	2.276006	TRUE	937.9963	0.921245
Eef1a1	6.0786	4.019872	62.35655	1.26E-278	1.9939	2.123556	TRUE	659.0762	0.59659
Lox	4.302448	0.886264	64.9806	8.06E-278	3.312941	3.519427	TRUE	615.7316	2.279349
Cthrc1	4.413912	0.849048	65.51004	4.98E-276	3.457993	3.671734	TRUE	601.7149	2.378139

In this table, you can select a list of genes as the candidate list. For example, order these genes by *P*-values and $\log_2$FC and select the top 400 genes.

[Step 3: Search a gene list of interest] SsIHDB provides batch search functionality. You can input a long list of gene symbols separated by commas, blanks, or enters (but not a combination of them) into the search box in the header. This will return all matched genes that are curated by ssIHDB and a table containing detailed information of these genes. At the top of the table, all matched genes and the total number of matched genes will be shown. Users can copy these genes for gene set enrichment analysis.

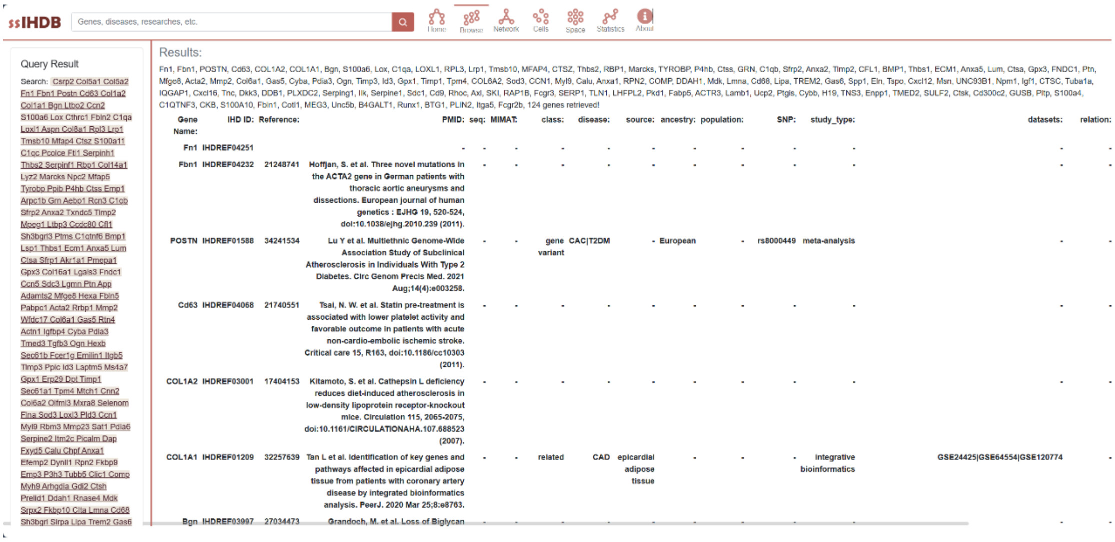

[Step 4: Construct a (neighbor graph) network from genes of interest] You can choose the whole returned gene list or a part of it to construct a network, or to find all primary neighbors of these genes in IHDKG. For example, to construct a network from all returned genes, you can click the "Network" button at the bottom of the page, and a network without other genes/drugs will be constructed. Besides, node centrality will be automatically calculated and could be downloaded by clicking the buttons on the left bottom of the page.

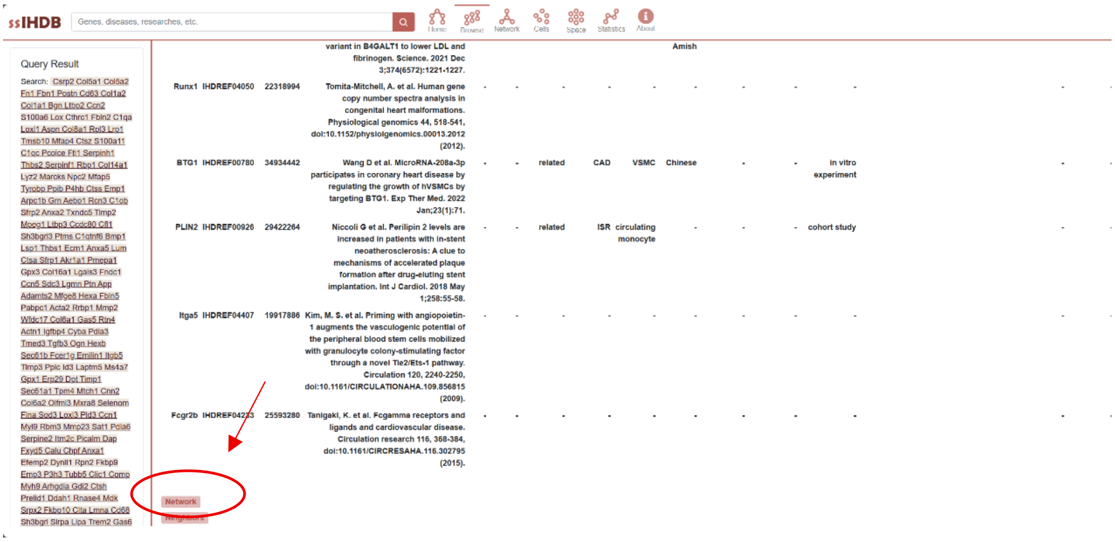

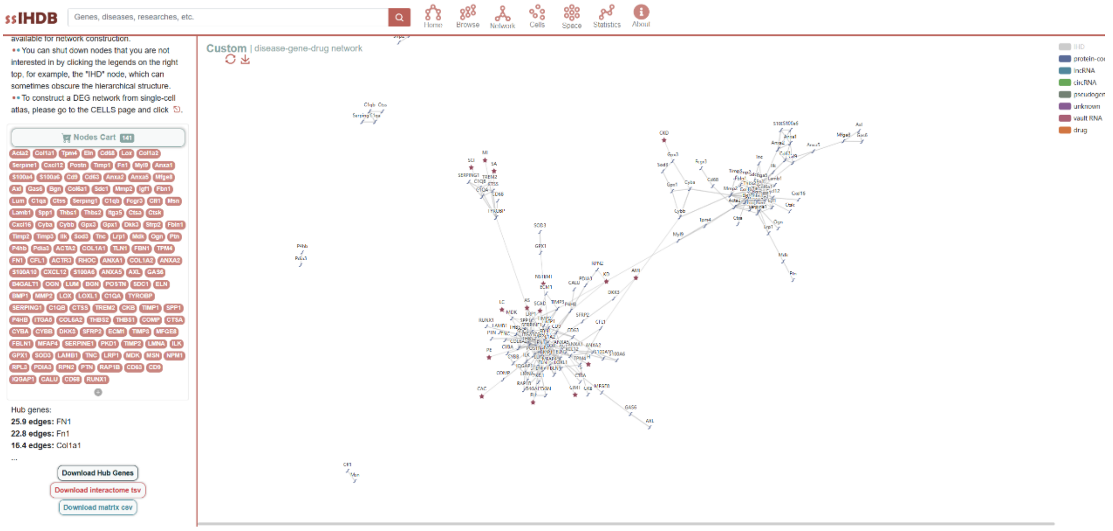

Otherwise, you can select a subset of these genes and click the "Neighbors" button to obtain all neighboring nodes, including protein-coding genes, non-coding genes, drugs, and comorbidities, and construct a large network. For example, we select Fndc1, Fstl1, Fn1, Runx1, Ddah1, Fbn1, and Postn here for neighbor graph network construction. Now users can inspect whether these genes were potential drug targets and find interacting proteins and miRNAs of them.

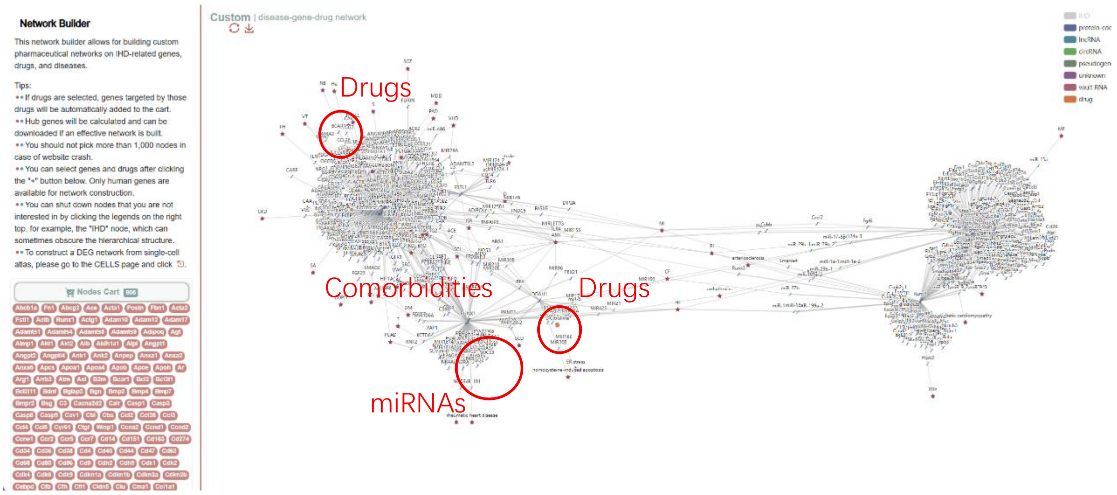

References

1 Kans J. Entrez Direct: E-utilities on the Unix Command Line. Available from: https://www.ncbi.nlm.nih.gov/books/NBK179288/.

2 Bird S, Edward Loper and Ewan Klein. Natural Language Processing with Python: O'Reilly Media Inc.; 2009.

3 Volders PJ, Anckaert J, Verheggen K, Nuytens J, Martens L, Mestdagh P, et al. LNCipedia 5: towards a reference set of human long non-coding RNAs. *Nucleic*

Acids Res 2019;**47**:D135-9.

4 Kozomara A, Birgaoanu M, Griffiths-Jones S. miRBase: from microRNA sequences to function. *Nucleic Acids Res* 2019;**47**:D155-D62.

5 Liu M, Wang Q, Shen J, Yang BB, Ding X. Circbank: a comprehensive database for circRNA with standard nomenclature. *RNA Biol* 2019;**16**:899-905.

6 Knox C, Wilson M, Klinger Christen M, Franklin M, Oler E, Wilson A, et al. DrugBank 6.0: the DrugBank Knowledgebase for 2024. *Nucleic Acids Res* 2024;**52**:D1265-75.

7 Szklarczyk D, Kirsch R, Koutrouli M, Nastou K, Mehryary F, Hachilif R, et al. The STRING database in 2023: protein–protein association networks and functional enrichment analyses for any sequenced genome of interest. *Nucleic Acids Res* 2023;**51**:D638-46.

8 Huang HY, Lin YC, Li J, Huang KY, Shrestha S, Hong HC, et al. miRTarBase 2020: updates to the experimentally validated microRNA–target interaction database. *Nucleic Acids Res* 2020;**48**:D148-54.

9 Wang P, Guo Q, Qi Y, Hao Y, Gao Y, Zhi H, et al. LncACTdb 3.0: an updated database of experimentally supported ceRNA interactions and personalized networks contributing to precision medicine. *Nucleic Acids Res* 2022;**50**:D183-9.

10 Bernstein NJ, Fong NL, Lam I, Roy MA, Hendrickson DG, Kelley DR. Solo: doublet identification in single-cell RNA-Seq *via* semi-supervised deep learning. *Cell Syst* 2020;**11**:95-101 e5.

11 Oliveira F, Bondareva O, Rodriguez-Aguilera JR, Sheikh BN. Cultured brain pericytes adopt an immature phenotype and require endothelial cells for expression of canonical markers and ECM genes. *Front Cell Neurosci* 2023;**17**:1165887.

12 Gavish A, Tyler M, Greenwald AC, Hoefflin R, Simkin D, Tschernichovsky R, et al. Hallmarks of transcriptional intratumour heterogeneity across a thousand tumours. *Nature* 2023;**618**:598-606.

13 Hao Y, Stuart T, Kowalski MH, Choudhary S, Hoffman P, Hartman A, et al. Dictionary learning for integrative, multimodal and scalable single-cell analysis. *Nat Biotechnol* 2024;**42**:293-304.

14 Dominguez Conde C, Xu C, Jarvis LB, Rainbow DB, Wells SB, Gomes T, et al. Cross-tissue immune cell analysis reveals tissue-specific features in humans.

Science 2022;**376**:eabl5197.
15 Nguyen HCT, Baik B, Yoon S, Park T, Nam D. Benchmarking integration of single-cell differential expression. *Nat Commun* 2023;**14**:1570.
16 Liu Z, Sun D, Wang C. Evaluation of cell-cell interaction methods by integrating single-cell RNA sequencing data with spatial information. *Genome Biol* 2022;**23**:218.
17 Dimitrov D, Schafer PSL, Farr E, Rodriguez-Mier P, Lobentanzer S, Badia IMP, et al. LIANA^{+} provides an all-in-one framework for cell-cell communication inference. *Nat Cell Biol* 2024;**26**:1613-22.
18 Shao X, Liao J, Li C, Lu X, Cheng J, Fan X. CellTalkDB: a manually curated database of ligand–receptor interactions in humans and mice. *Brief Bioinform* 2021;**22**:bbaa269.
19 Liang Q, Huang Y, He S, Chen K. Pathway centric analysis for single-cell RNA-seq and spatial transcriptomics data with GSDensity. *Nat Commun* 2023;**14**:8416.
20 Gayoso A, Lopez R, Xing G, Boyeau P, Valiollah Pour Amiri V, Hong J, et al. A Python library for probabilistic analysis of single-cell omics data. *Nat Biotechnol* 2022;**40**:163-6.
21 Yu G, Wang LG, Han Y, He QY. clusterProfiler: an R package for comparing biological themes among gene clusters. *OMICS* 2012;**16**:284-7.
22 Hu Y, Xie M, Li Y, Rao M, Shen W, Luo C, et al. Benchmarking clustering, alignment, and integration methods for spatial transcriptomics. *Genome Biol* 2024;**25**:212.
23 Kleshchevnikov V, Shmatko A, Dann E, Aivazidis A, King HW, Li T, et al. Cell2location maps fine-grained cell types in spatial transcriptomics. *Nat Biotechnol* 2022;**40**:661-71.
24 Kuppe C, Ramirez Flores RO, Li Z, Hayat S, Levinson RT, Liao X, et al. Spatial multi-omic map of human myocardial infarction. *Nature* 2022;**608**:766-77.
25 Mathieu Bastian SH, Mathieu Jacomy. Gephi: an open source software for exploring and manipulating networks. *Proceedings of the International AAAI Conference on Web and Social Media* 2009;**3**:361-2.
